# Disease characterization in liquid biopsy from HER2-mutated, non-amplified metastatic breast cancer patients treated with neratinib

**DOI:** 10.1038/s41523-022-00390-5

**Published:** 2022-02-18

**Authors:** Stephanie N. Shishido, Rahul Masson, Liya Xu, Lisa Welter, Rishvanth Kaliappan Prabakar, Anishka D’ Souza, Darcy Spicer, Irene Kang, Priya Jayachandran, James Hicks, Janice Lu, Peter Kuhn

**Affiliations:** 1 Convergent Science Institute in Cancer (CSI-Cancer), Michelson Center for Convergent Bioscience, University of Southern California,1002 Childs Way, MCB 220, Los Angeles, CA 90089 USA; 2 USC Norris Comprehensive Cancer Center, Keck School of Medicine, University of Southern California, 1441 Eastlake Ave, NTT-3440, Los Angeles, CA 90033 USA

**Keywords:** Cancer genomics, Metastasis, Tumour heterogeneity, Breast cancer, Tumour biomarkers

## Abstract

Metastatic breast cancer (mBC) patients have a high risk of progression and face poor prognosis overall, with about one third (34%) surviving five years or more. In rare instances (2–4% of cases) patients with mBC have *ERBB2* (HER2) activating mutations but are *ERBB2* non-amplified. Neratinib is a potent, irreversible inhibitor that binds HER2 and inhibits downstream signaling. We used the previously validated high-definition single cell assay (HDSCA) workflow to investigate the clinical significance of the liquid biopsy in ERBB2 mutant, non-amplified, post-menopausal mBC patients starting neratinib and fulvestrant combination therapy. Characterization with a comprehensive liquid biopsy methodology (HDSCA) included genomic analysis of both the cell-free DNA (cfDNA) and single circulating tumor cells (CTCs) to monitor tumor evolution and identify potential mutational variants unique to the patient’s clinical response. A limited series of five sequentially enrolled patients presented here were from the MutHER (https://www.clinicaltrials.gov, NCT01670877) or SUMMIT (https://www.clinicaltrials.gov, NCT01953926) trials. Patients had an average of 5.4 lines of therapy before enrollment, variable hormone receptor status, and *ERBB2* mutations at diagnosis and during treatment. CTC enumeration alone was not sufficient to predict clinical response. Treatment pressure was shown to lead to an observable change in CTC morphology and genomic instability (GI), suggesting these parameters may inform prognosis. Single cell copy number alteration (CNA) analysis indicated that the persistence or development of a clonal population of CTCs during treatment was associated with a worse response. Hierarchical clustering analysis of the single cells across all patients and timepoints identified distinct aberrant regions shared among patients, comprised of 26 genes that are similarly affected and may be related to drug resistance. Additionally, the genomic analysis of the cfDNA, identified new mutations in *ERBB2*, *PIK3CA*, and *TP53* that arose likely due to treatment pressure in a patient with poor response, further providing insights on the dynamics of the cancer genome over the course of therapy. The data presented in this small cohort study demonstrates the feasibility of real-time molecular profiling of the cellular and acellular fractions of the liquid biopsy using the HDSCA methodology. Additional studies are necessary to determine the potential use of morphometric and genomic analysis as a prognostic tool to advance personalized oncology.

## Introduction

Breast cancer (BC) is the most common cancer in women worldwide, accounting for 24.5% of all newly diagnosed cancer cases^1^. Distant metastatic lesions, including de novo metastases and distant recurrence, represent the most advanced form of this disease and are the primary drivers of lethality. Metastatic BC (mBC) patients have a high risk of progression and face poor prognosis overall, with only about one third (34%) surviving 5 years or more^2^. Amplification of the epidermal growth factor 2 (*ERBB2*) gene, which encodes the human epidermal growth factor receptor 2 (HER2) protein, has been implicated in several malignancies, most prominently BC, and is a well-established therapeutic target^3–7^. *ERBB2* amplification has been associated with an aggressive BC phenotype and poor disease-free survival^4^. Despite advances in HER2-targeted therapies, some patients remain unresponsive or develop resistance^8^. The small molecule neratinib (Puma Biotechnology, Los Angeles, CA, USA) was developed to overcome this resistance by targeting the HER2 signaling cascade using a mechanism of action different than the monoclonal antibodies trastuzumab and pertuzumab. Neratinib is an oral, irreversible HER2/EGFR tyrosine kinase inhibitor that covalently binds the intracellular tyrosine kinase domain to prevent autophosphorylation and downstream signaling^9–12^. In Phase I and II studies, single agent neratinib therapy was well tolerated by patients, reducing the overall tumor burden and prolonging progression free survival (PFS) in patients with known *ERBB2* or *ERBB1/EGFR* positive breast or lung tumors^9,11^.

In rare instances, patients with mBC have HER2 activating mutations but are clinically considered HER2 negative (non-amplified by FISH). *ERBB2* mutations that activate the HER2 signaling cascade by affecting the extracellular domain, transmembrane domain, or tyrosine kinase domain of the HER2 protein are considered activating mutations, which may occur in the presence of normal HER2 expression levels^13–15^. Preclinical data indicates that *ERBB2* mutations may confer a sensitivity to HER2-targeted drugs and that activating HER2 mutations may drive cancer progression similarly to *ERBB2* amplification^16^. Patients that have *ERBB2* mutation in the absence of gene amplification account for only 2–4% of all metastatic BC cases^13^. BC patients without an *ERBB2* amplification do not typically respond to the recombinant monoclonal antibody trastuzumab likely due to the lower levels of receptor expression and, hence, reduced efficacy^17^. Importantly, neratinib has been found to have clinical activity on heavily pre-treated *ERBB2* mutant, non-amplified mBC, with a clinical benefit rate of 36%^18^. Two Phase II trials have been established to assess the efficacy of neratinib plus fulvestrant combination therapy in patients with HER2-negative mBC that carry an *ERBB2* mutation due to the predominance of estrogen receptor (ER) positivity in this subset of patients. The MutHER trial (https://www.clinicaltrials.gov, NCT01670877), a Phase II study investigating neratinib alone and in combination with fulvestrant in metastatic HER2 non-amplified, HER2 mutant BC was launched in 2012. This is a non-randomized, interventional study consisting of 56 participants enrolled at 17 study locations. The Neratinib HER mutation basket study named SUMMIT (https://www.clinicaltrials.gov, NCT01953926) was subsequently launched in 2013. This is an ongoing, open-label, non-randomized, multicenter, multinational, Phase II study exploring the efficacy and safety of neratinib as a monotherapy or in combination with other therapies in patients with *ERBB* mutation-positive or *EGFR* gene-amplified solid tumors. This trial has an estimated enrollment of 650 participants, recruiting across 52 study locations.

Clinical trial data around neratinib indicates initial clinical benefit for patients with HER-positive (immunohistochemistry or FISH) or *ERBB1/2* mutated, advanced solid cancers, but there is a lack of knowledge around the mechanism of neratinib resistance. In a study looking at neratinib resistance, drug-resistant BC cells had a reduced expression of all EGFR family members, including HER3 which is not targeted by neratinib, but heterodimerizes with HER2^19,20^. This is contrary to other HER2 targeting drugs such as lapatinib and trastuzumab, in which EGFR and HER2 are upregulated^21–23^, suggesting neratinib may have alternative pathways of resistance. This warrants a methodology to monitor the evolution of resistance or lack of treatment efficacy in real time.

Knowledge of the cancer subtype, its treatment history, and genomic profile at the time of decision making are essential for selection of therapy and development of new therapeutics to prolong survival and reduce mortality rates. A comprehensive measure of the liquid biopsy may assist in solving complex clinical problems by tracking cellular evolution and drug resistance, revealing agents that are not efficacious, as well as developing a stratification system in order to avoid inappropriate or excessive therapeutic use. The presence of circulating tumor cells (CTCs) has been associated with reduced survival in patients with metastatic carcinomas^24–29^. Determination of the genetic components of treatment resistance (resistant genotypes) and their correlation to resistant phenotypes can potentially promote clinical application of CTCs. Plasma circulating cell-free DNA (cfDNA) has also been reported in many studies as a potential prognostic tool for mutational analysis and personalized cancer treatment^30–33^. Genomic analysis of both CTCs and cfDNA have significant potential value for both the clinical and research communities.

In a small, single site cohort of post-menopausal patients with metastatic *ERBB2* mutant/non-amplified BC receiving neratinib and fulvestrant from the studies described above, peripheral blood (PB) samples were collected as liquid biopsies during treatment and analyzed to characterize CTCs and cfDNA. We used the previously validated high-definition single cell assay (HDSCA) workflow to investigate the clinical significance of CTCs in *ERBB2* mutant, non-amplified BC patients starting neratinib and fulvestrant combination therapy. The HDSCA workflow is an enrichment-free approach used to identify high-definition CTCs by classifying all nucleated cells in the PB according to various morphological parameters and the cellular expression of leukocyte and epithelial markers to detect rare epithelial cells in circulation^34–36^. Genomic analysis of the cfDNA and individual CTCs was conducted to monitor tumor evolution and identify potential mutational variants unique to the patient’s clinical response while receiving combined neratinib and fulvestrant treatment.

Utilizing a comprehensive liquid biopsy (CTCs and cfDNA) for genomic characterization, we have identified pre-existing and acquired alterations relevant to the disease state and potential treatment response. This type of comprehensive characterization of the genomic landscape may guide clinicians to identify the most effective therapies for individual cancer patients. The HDSCA workflow may be utilized as a prognostic tool for therapeutic response to directly impact treatment decisions for metastatic BC patients with *ERBB2* mutations.

## Results

### Patient demographics

**Fig. 1:**
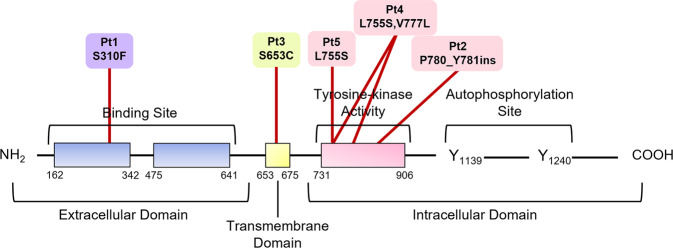
Patient specific ERBB2 mutations identified in the tumor at the time of enrollment. Patients 1 and 2 had pathological assessments conducted by Foundation One, while Patients 3–5 had pathological assessment conducted by CARIS. Purple: Excellent responder, Pink: Average responder, Yellow: Poor responder.

Five patients with metastatic BC from the MutHER or SUMMIT trials had PB samples collected for liquid biopsy analysis using the HDSCA workflow. Multiple draws were collected for each patient, allowing for a total of 24 PB samples to be evaluated. All five patients had HER2 negative metastatic BC at diagnosis. Demographics, clinical disease characteristics, and *ERBB2* mutation identified in the tumor of each patient are provided in Table 1. At enrollment Patients 2, 4, and 5 had mutations in the intracellular kinase domain of *ERBB2*. Patient 1 had a mutation in the extracellular Furin-like domain, while Patient 3 had a mutation in the transmembrane domain of *ERBB2*. Patients had variable hormone receptor status at diagnosis and at recurrence. Interestingly, the survival outcomes were associated with the region of *ERBB2* affected (Fig. 1). All average responders, Patients 2, 4, and 5, had a mutation in the intracellular kinase domain. Patient 1, the best responder, had a mutation in the extracellular domain, while patient 3, the worst responder, had a mutation in the transmembrane domain.

**Table 1:** Patient demographics and clinical disease characteristics.

	Patient#1	Patient#2	Patient#3	Patient#4	Patient#5
Race	Caucasian	Caucasian	Caucasian	Caucasian	Caucasian
Age	58	61	57	85	59
# of prior Tx	7	11	3	4	2
Histologic Type	Lobular	Ductal	Lobular	Ductal	Ductal
ER status at Dx	Positive	HR+	Positive	Positive	Positive
PR status at Dx	Positive	HR+	Positive	Negative	Positive
HER2 status at Dx	Negative	Negative	Negative	Negative	Negative
ER status at Recurrence	Negative	N/A	Positive	Positive	N/A
PR status at Recurrence	Positive	N/A	Negative	Negative	N/A
Date Started Neratinib	6/29/2016	10/4/2016	10/31/2016	2/22/2017	8/23/2017
CEA at start of Tx	2.20	38.50	31.90	17	N/A
CA27.29 at start of Tx	102.80	N/A	3735.00	N/A	N/A
Last imaging scan	1/16/2018	5/18/2017	12/21/2016	1/3/2018	4/3/2018
Response on last scan	PD	PD	PD	PD	PD
Days to progression	566	226	515	315	224
ERBB2 mutation	S310F	P780_Y781insGSp	S653C	V777L, L755S	L755S
ERBB2 region	Furin-like domain	Kinase domain	Transmembrane domain	Kinase domain	Kinase domain
Mutation type	Missense	Inframe insertion	Missense	Missense	Missense
# of samples analyzed	10	6	2	4	2
Tumor Pathology Report	ERBB2 (S310F), ARID1A (Q479^a^), CDH1 (l581FS^a^1), NOTCH2 (SEC22B-NOTCH fusion)	ERBB2 (P780_Y781ins), ARID1A (Q1172^a^), CDH1 (L585fs^a^4), NUP93 (E14K), TBX3 (Y248fx^a^11); Amplified: CCND11, EMSY, FGF19, FGF3, FGF4	ERBB2 (S653C), PIK3CA (E545K), PTPN11 (E76K – subclonal); NF1 rearrangement of exon 37; ARID1A rearrangement intron 1; Amplified: CCND1, FGFR1, FGF19, FGF3, FGF4, 2NF703	ERBB2 (L755S, V777L), PIK3CA (H1047A); Amplified: MDM2; IHC: ERBB2-, PD-1-, PTLN-, TrkA/B/C-, PR- AR+, ER+, ERCC1+, PTEN+	ERBB2 (L755S); CISH: cMET, TOP2A, and HER2/neu not amplified; IHC: ALK-, cMET-, EGFR-, ERCC1-, HER2-, MGMT-, PGP-, PTEN-, TOPO1-, TS-, TUBB3-, AR+, ER+, PD-1+, PD-L1+, PR+, TLE3+

*Tx* Treatment, *ER* Estrogen receptor, *PR* Progesterone receptor, *HR* Hormone receptor, *Dx* Diagnosis, *NA* Not available, *SD* Stable disease, *PD* Progressive disease.

^a^Clinical information provided did not specify ER or PR.

Patients had a variable number of PB draws depending on treatment and outcome. All samples were processed through the HDSCA workflow as described in the methods. This workflow has been extensively validated in several large studies^36–38^ and has been employed in developing a clinically useful assay in commercial production^39–42^. A detailed description of the assay and validation is presented in the Supplemental Information.

This is the first study of a comprehensive liquid biopsy in this disease context and is limited in regard to the number of patients analyzed. Therefore, each patient is presented individually with a multianalyte profile and carefully interpreted with appropriate caution to enhance the reader’s knowledge and attempt to elucidate a mechanism of resistance.

### Patient 1

Patient 1 was diagnosed in 2005 with localized BC that was subsequently resected and had since progressed twice prior to enrollment in the MutHER trial. Prior metastatic lesions were detected in the bone, liver, and lung. A total of 10 samples were collected post therapy starting at day 113, with follow-up draws collected on days 139, 167, 195, 222, 285, 314, 341, and 371. Stable disease status was determined prior to draw 10, while progressive disease was reported 140 days post draw 10. Unfortunately, no additional PB samples were collected during this time frame. Patient 1 was an excellent responder to treatment with a PFS of 566 days.

**Fig. 2:**
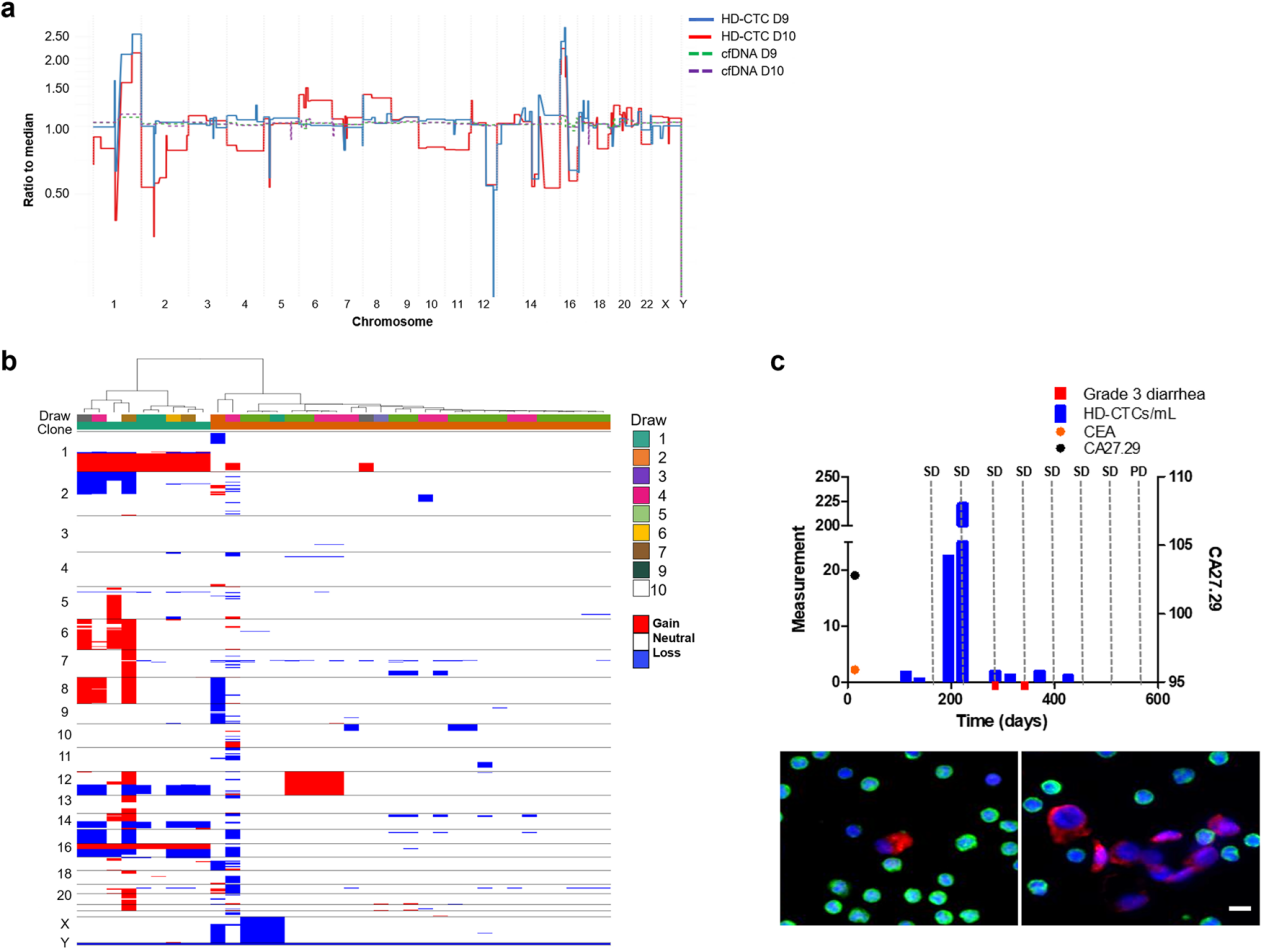
Comprehensive analysis of the liquid biopsy for Patient 1. **a** Similar clonal architecture identified in the CTCs was also detectable in the cfDNA, with a greater tumor fraction present in follow-up sampling. **b** Heatmap and phylogenic tree of single cell CNAs across the entire population of cells isolated. Draw number and clones are identified using color key. **c** Longitudinal analysis of CTCs and tumor antigen burden in Patient 1. Clinical features include CEA and CA27.29 measurements taken periodically over the course of treatment. SD stable disease, PD progressive disease. Representative images of CTCs taken at 400X. Scale bar = 10 µm. White: ER, Red: CK, Green: CD45, Blue: DAPI.

The cfDNA from Patient 1 samples were analyzed, but the tumor fraction was low to nondetectable by CNA profiling in the majority of draws, suggesting the ctDNA represented less than ~5% of the total cfDNA. The fraction of ctDNA and GI score of each cfDNA sample is provided in Supplemental Table 3. The ctDNA fraction was calculated using two methods which showed general agreement across samples. Draws 9 and 10 were determined to have approximately 10 and 17% tumor fraction, respectively, both of which show a gain in chromosome 1q, similar to the CTCs identified in those draws (Fig. 2a). The cfDNA GI score for Patient 1 over the course of treatment remained roughly constant with a median of 13.82 (range 10.17–15.53, mean 14.87). Further deep sequencing using the Ion Torrent BC hotspot panel was conducted on cfDNA from draws 4, 8, 9, and 10. An increase in the fraction of detectable mutation in *TP53* mutation (R213*) was observed during treatment (0.08–4.5%; Table 2). Additionally, the *ERBB2* mutation (S310F) identified from the tumor pathology at the start of therapy was detectable in draws 9 and 10, but not in draws 4 and 8. No additional hotspot mutations were identified in the cfDNA.

**Table 2:** Mutational analysis of cfDNA.

Patient#1	Gene	Draw 4	Draw 8	Draw 9	Draw 10
	*ERBB2*	–	–	S310F (0.18%)	S310F (0.29%)
	*TP53*	R213* (0.08%)	R213* (0.47%)	R213* (2.91%)	R213* (4.5%)
Patient#2	Gene	Draw 4	Draw 6		
	*ERBB2*	–	G776V (1.94%)		
Patient#3	Gene	Draw 1	Draw 2		
	*ERBB2*	–	L755S (0.13%)		
	*ESR1*	E380Q (0.50%), Y537N (0.33%)	E380Q (0.07%)		
	*PIK3CA*	E545K (2.7%)	E545K (30%), E726K (0.09%)		
	*TP53*	R248Q (0.31%)	R248Q (0.19%), G245D (0.11%)		
Patient#4	Gene	Draw 1	Draw 2	Draw 4	
	*ERBB2*	L755S (0.79%), V777L (0.93%)	–	L755S (0.78%), V777L (0.46%)	
	*PIK3CA*	H1047R (1.03%)	–	H1047R (0.58%)	
	*TP53*	H365fs (0.38%)	–	–	
Patient#5	Gene	Draw 1			
	*ERBB2*	L755S (14.39%)			

Oncomine cfDNA Assay v2 detects hotspot mutations in the cfDNA of BC patients. (−): no mutations detected. Variant allele frequency is provided in parentheses (%).

*Indicates a termination codon.

Patient 1 had stable CTC kinetics from draw 1 to draw 3 ranging between 0–1.09 CTCs/ml, at which times the patient had stable disease (Fig. 2c). Subsequent draws 4 and 5 showed an increase in the number of CTCs detected, with a maximum of 222.35 CTCs/ml at draw 5. At draw 6 the CTC count decreased to 0 CTCs/ml. CTC kinetics remained stable to draw 10, ranging from 0–1.30 CTCs/ml. The CTCs displayed a heterogeneous morphology wherein nuclear size and CK signal intensity varied greatly.

In parallel with the cfDNA, single cell genomic analysis was conducted on CTCs. CNA analysis of 54 CTCs was conducted from samples collected from all draws. A total of 33 (61%) CTCs were identified to have genomic alterations (Fig. 2b). There were no CTCs detected in draw 8 and thus single cell genomic analysis was not conducted. In draw 1, only a few CTCs were detected with GI scores ranging from 182.95 to 228.99. A single CTC was identified in draw 2 with a GI score of 318.30. Hierarchical cluster analysis showed that this CTC was similar to two highly aberrant cells from draw 4 (GI score of 721.97 and 604.97). These deep alterations may be due to homologous recombination, leading to the loss or gain of one complete arm of a chromosome. The remaining CTCs from draw 4 were determined to represent 2 subclones that were also present in draw 5. One clone had losses at chromosome 7q (*SMO, BRAF*) and 14q (*NKX2-1, FOXA1*), while the other had gains in chromosome 12 (*CCND2, KRAS, CDK4, PTPN11*). Draw 5 had one unique subclone with losses at 10p (*GATA3, KIF5B, ABl1*) only found at this timepoint, and another subclone with losses on the X chromosome (*ELF4, PHF6, RPL10*) that clustered with a single cell found in draw 1. Both of these subclones were eliminated from circulation as treatment continued, suggesting treatment sensitivity. Very few cells were identified in draws 6–10. With the exception of one cell from draw 9, all the CTCs identified from draws 6–10 were determined to be clonal with gains at chromosome 1q and 16p, and losses at 16q, 12q, and 14q (GI score range: 459.9–906.75). These cells clustered with single cells from draw 4 and draw 1. Genomic analysis of single cells suggests that a dominant subclone in draw 1 was resistant to treatment and maintained, becoming more present in circulation as treatment persisted (Supplemental Fig. 1).

### Patient 2

Patient 2 was diagnosed in 2001 with localized BC that was subsequently resected and had progressed five times prior to enrollment in the MutHER trial. Metastatic lesions were detected in the bone prior to enrollment. This patient was on trial for 226 days prior to death from multi-organ failure. Tumor status at the time of death is unknown. Patient had stable disease at last follow-up visit. A total of six samples were collected post therapy starting at day 29, with follow-up draws at days 57, 120, 148, 176, and 211. The patient was determined to have progressive disease 15 days after the last PB collection and was an average responder to treatment.

**Fig. 3:**
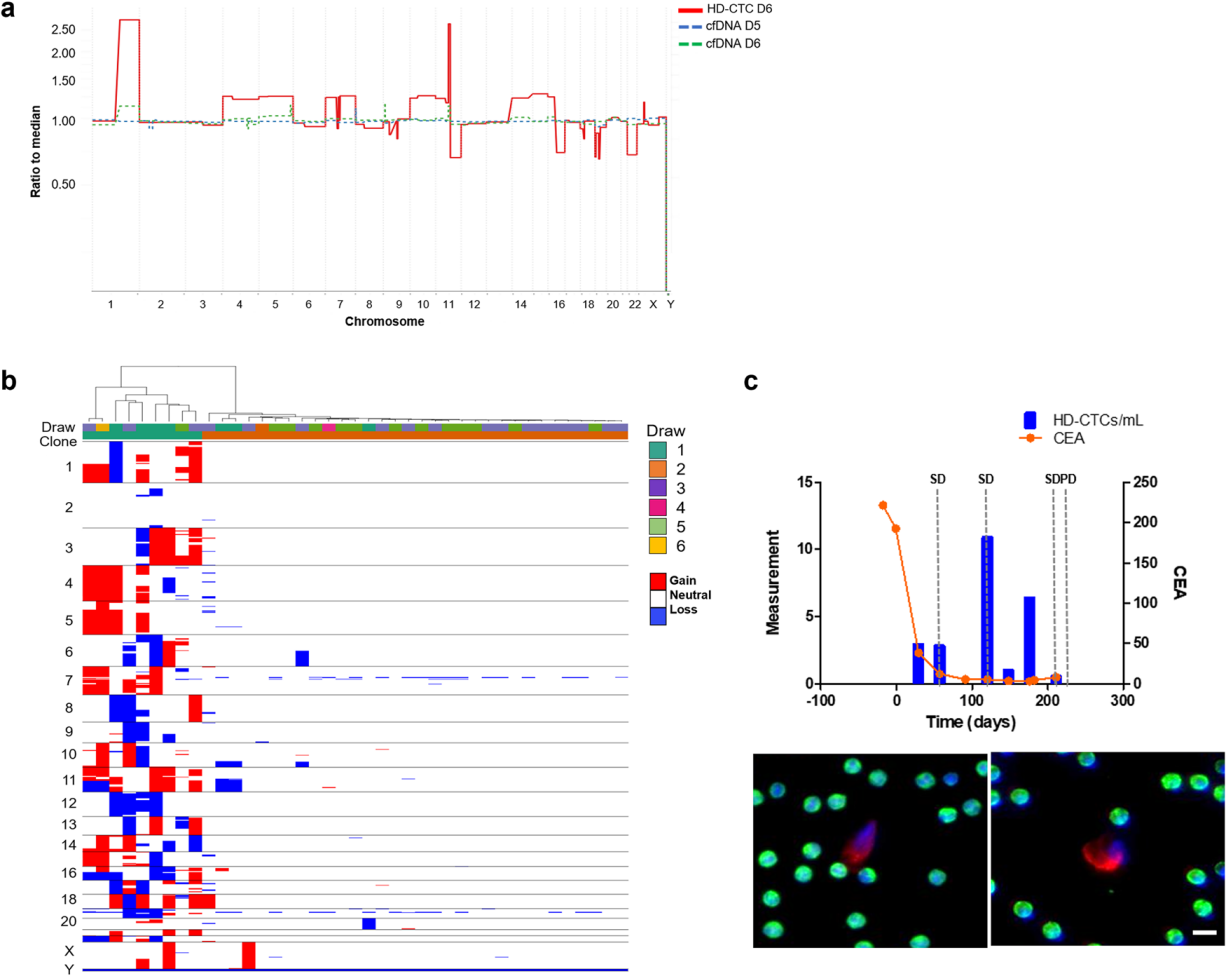
Comprehensive analysis of the liquid biopsy for Patient 2. **a** Similar clonal architecture identified in the CTCs was also detectable in the cfDNA, with a greater tumor fraction present in follow-up sampling. **b** Heatmap and phylogenic tree of single cell CNAs across the entire population of cells isolated. Draw number and clones are identified using color key. **c** Longitudinal analysis of CTCs and tumor antigen burden in Patient Clinical features include CEA measurements taken periodically over the course of treatment. SD stable disease, PD progressive disease. Representative images of CTCs taken at 400X. Scale bar = 10 µm. White: ER, Red: CK, Green: CD45, Blue: DAPI.

Patient 2 was determined to have a low percent of tumor fraction (<10%) though the cfDNA GI score increased from an initial 11.06 at draw 1 to 33.25 at draw 6 (median 14.83, range 6.7–33.25, mean 17.50). Interestingly there is an observable gain in chromosome 1q at draw 5 and draw 6 (Fig. 3a). Draws 4 and 6 showed the gain of a new *ERBB2* mutation (G776V) in the tyrosine kinase intracellular domain (Table 2) which was not identified in the tumor pathology at the start of therapy. The *ERBB2* mutation (P780_Y781ins) identified in the tumor pathology was not found in the cfDNA. No additional hotspot mutations were identified in the cfDNA.

Patient 2 presented with 1.93 CTCs/ml in draw 1 with stable kinetics to draw 2 (Fig. 3c). There was an increase to 10.87 CTCs/ml at draw 3 in which stable disease was confirmed. At draw 4 there was a decrease in the number of cells detected to 0.96 CTCs/ml, followed by a subsequent increase to 6.37 CTCs/ml in draw 5. Draw 6 was the last sample collected for this patient, in which 0.53 CTCs/ml were detected and stable disease confirmed. Progressive disease was reported 15 days after this timepoint. Patient 2 had a heterogeneous population of cells, in which nuclear size and CK signal intensity varied greatly.

CNA analysis was conducted on 41 cells isolated from Patient 2 samples. Only, 11 (26.8%) CTCs were identified to have genomic alterations but lacked clonality (Fig. 3b). CTCs from draw 1 were highly aberrant with GI scores ranging from 465.78–1983.79. The single cell detected in draw 2 had minimal to no alterations. Draw 3 had many detectable CTCs, but only 2 (18%) were highly aberrant with multiple alterations. The single cell detected in draw 4 had minimal to no alterations. Interestingly in draw 5, one of the two CTCs detected was aberrant (GI score of 235.3) while the other was flat (GI score of 17.5). A single CTC was detected in Draw 6 and identified to have a similar genomic architecture to that of the CNA profile for the cfDNA (Fig. 3a) as well as 1 CTC from Draw 3. Overall, the CTCs detected from Patient 2 indicate high tumor heterogeneity suggestive of increasingly active disease and a lack of detectable lineage evolution during the course of treatment.

### Patient 3

Patient 3 was diagnosed in 2015 with localized BC that was subsequently resected and had since progressed three times prior to enrollment in the SUMMIT trial. Prior metastatic lesions were detected in the axillary lymph nodes, omentum, liver, and lung. This patient was on trial for 51 days prior to progression of disease. Two samples were collected post therapy initiation starting at day 15 with a follow-up sample at day 51.

**Fig. 4:**
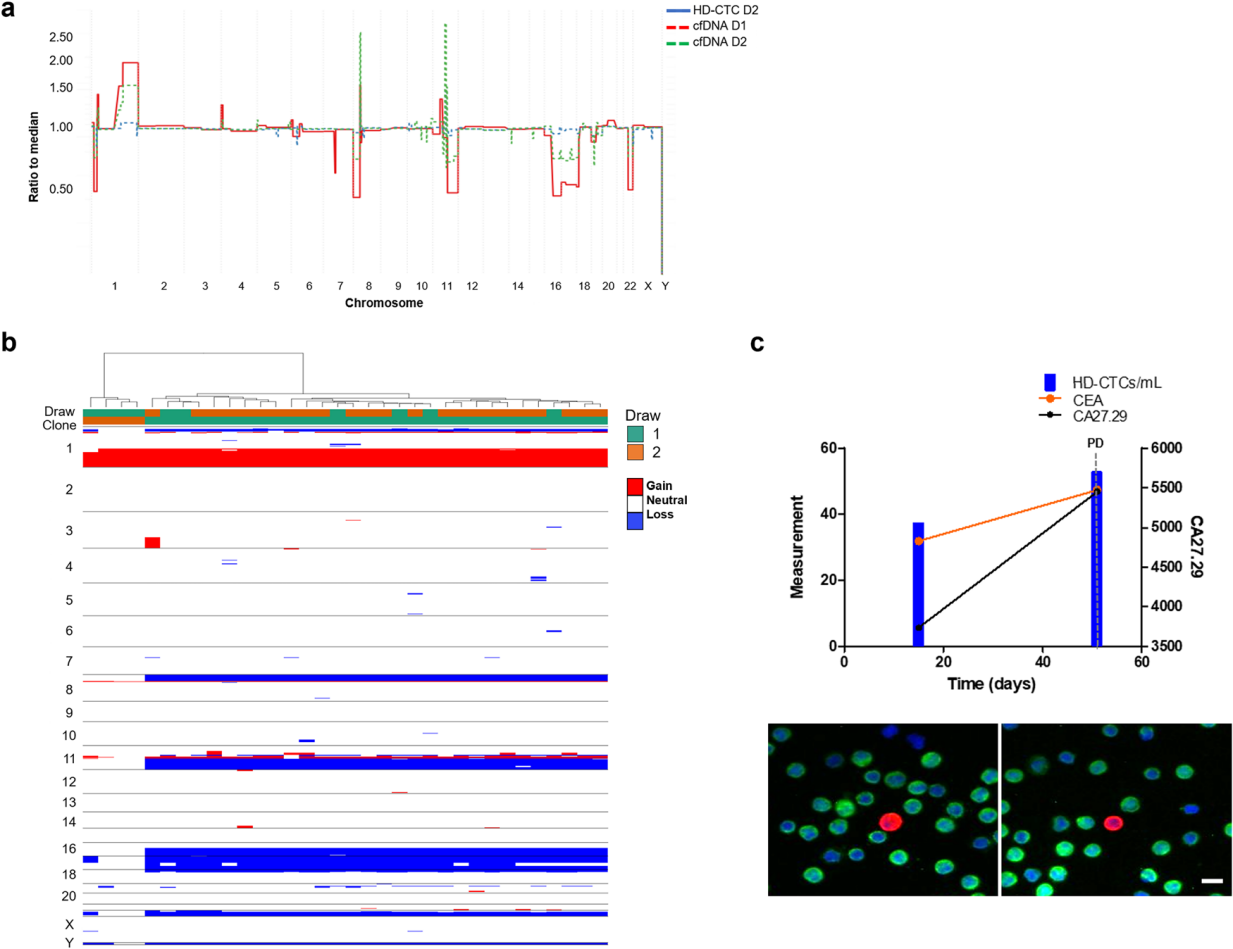
Comprehensive analysis of the liquid biopsy for Patient 3. **a** Similar clonal architecture identified in the CTCs was also detectable in the cfDNA, with a greater tumor fraction present in follow-up sampling. **b** Heatmap and phylogenic tree of single cell CNAs across the entire population of cells isolated. Draw number and clones are identified using color key. **c** Longitudinal analysis of CTCs and tumor antigen burden in Patient 3. Clinical features include CEA and CA27.29 measurements taken periodically over the course of treatment. PD progressive disease. Representative images of CTCs taken at 400X. Scale bar = 10 µm. White: ER, Red: CK, Green: CD45, Blue: DAPI.

The cfDNA from Patient 3 was identified to have an aberrant CNA profile with a greater tumor fraction present in follow-up sampling (Fig. 4a). Draw 1 had approximately 7% tumor fraction, while draw 2 had 63% tumor fraction. The cfDNA GI score increased from 16.46 at draw 1 to 172.8 at draw 2. SNV analysis was conducted on both draws from Patient 3. The *ERBB2* mutation (S653C) identified in the tumor pathology prior to therapy was not part of the hotspot panel and was therefore not detectable. Mutations in *ERS1, PIK3CA*, and *TP53* were identified in both draws (Table 2). There was an observational increase in the fraction of *PIK3CA* (E454K) from 2.7% to 30% over the course of treatment. This mutation was also identified in the tumor pathology report prior to initiation of therapy. Additionally, at draw 2, Patient 3 presented with a new mutation in *ERBB2* (L755S) located in the tyrosine kinase intracellular domain, as well as new mutations in *PIK3CA* (E726K) and *TP53* (G245D).

At baseline, this patient presented with 37.08 CTCs/ml with a subsequent increase to 52.49 CTCs/ml after the start of therapy with confirmed disease progression (Fig. 4c). Patient 3 was taken off trial after 51 days and was considered a poor responder to treatment. All cells isolated from Patient 3 were determined to comprise a clonal population of CTCs that persisted in circulation despite treatment (Fig. 4b). This clonal architecture was also identified in the cfDNA. The major aberrations included gains in 1q (*ABL2, CDC73, ELK4*), 8p (*FGFR1*), and 11q (*CCND1*), and losses in 8p (*PCM1, WRN*), 11q (*ATM, SDHD, ARHGEF12*), 16q (*CYLD, CDH1*), 17 (*TP53, BRCA1*), and 22q (*CHEK2, PDGFB*). The CTCs from Patient 3 had a median GI score of 533.3 and a range of 67.7–606.6 (mean = 426.5). The chromosomal regions affected are common to both ductal and lobular hormone positive BC^43,44^ and consistent with loss of cell cycle control. Interestingly, in draw 1, four cells showed variant CNV profiles with fewer events that are consistent with evolutionary precursors to the main clone. This suggests that the gains at chromosome 1q, 8p, and 11p originated earlier in the cellular lineage than the losses, but that the losses confer an advantage to the cell.

### Patient 4

Patient 4 was diagnosed in 2014 with mBC and had progressed three times prior to enrollment in the SUMMIT trial. Prior metastatic lesions were detected in the liver. This patient was on trial for 314 days prior to progression of disease, representing an average responder to treatment in this cohort. A total of four samples were collected starting at day 0 prior to therapy and follow-up samples taken at days 14, 34, and 315 post therapy initiation. This is the only patient in this small cohort that has both primary and metastatic tumors present at the time of enrollment.

**Fig. 5:**
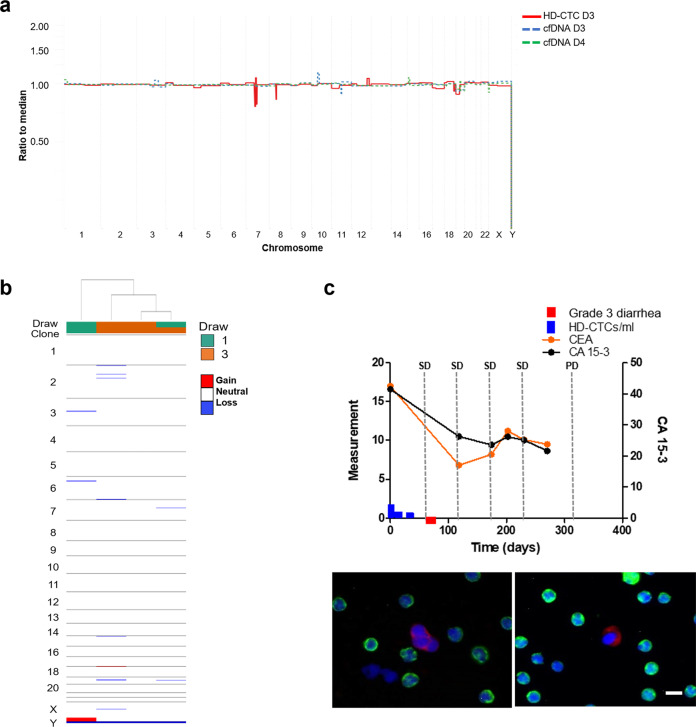
Comprehensive analysis of the liquid biopsy for Patient 4. **a** Similar clonal architecture identified in the CTCs was also detectable in the cfDNA, with a greater tumor fraction present in follow-up sampling. **b** Heatmap and phylogenic tree of single cell CNAs across the entire population of cells isolated. Draw number and clones are identified using color key. **c** Longitudinal analysis of CTCs and tumor antigen burden in Patient 4. Clinical features include CEA and CA15-3 measurements taken periodically over the course of treatment. SD stable disease, PD progressive disease. Representative images of CTCs taken at 400X. Scale bar = 10 µm. White: ER, Red: CK, Green: CD45, Blue: DAPI.

Patient 4 cfDNA samples had a low to nondetectable tumor fraction (Fig. 5a). Over the course of treatment, the cfDNA GI score decreased from 23.94 at draw 1–8.39 at draw 4 (median 17.75, range 8.39–23.94, mean 16.96). Draws 1, 2 and 4 were further analyzed for SNV (Table 2). There were no hotspot mutations detectable in draw 2. The *ERBB2* mutations (L755S, V777L) identified in the tumor pathology prior to treatment were detected in draws 1 and 4. Additional mutations in *PIK3CA* and *TP53* were detected at low frequency in draw 1 prior to therapy initiation.

Very few CTCs were detected in samples from Patients 4 (Fig. 5c). This is not surprising considering the low prevalence tumor fraction in the cfDNA. Patient 4 presented with 1.65 CTCs/ml in draw 1 with stable kinetics through draw 4, at which time this patient was determined to have progressive disease. From the two CTCs that were isolated for genomic analysis from Patient 4, minimal to no alterations were identified (Fig. 5b). The CTC isolated from draw 1 had a GI score of 80.6, while the CTC from draw 3 appeared flat with an GI score of 16.1. Together this suggests the circulation of genomically heterogenous CTCs potentially indicative of tumor heterogeneity.

### Patient 5

Patient 5 was diagnosed in 2008 with localized BC that was resected. Lesions were identified in the axillary lymph nodes in 2015, leading to a mastectomy and two subsequent treatment regimens prior to bone progression and enrollment in the SUMMIT trial. This patient was on trial for 224 days prior to progression of disease and considered an average responder to treatment. Two samples were collected starting at day 0 prior to therapy with a follow-up sample collected at day 224 post initiation of therapy.

The cfDNA CNA patterns reveal the extent of tumor heterogeneity and may add insight into treatment response or resistance. The cfDNA tumor fraction decreased from 14% to approximately 10% from draw 1 to draw 2. Similarly, the GI score decreased from an initial 46.28 at draw 1 to 15.88 at draw 2. Patient 5 draw 1 was analyzed for hotspot mutations using the cfDNA (Table 2). Unfortunately, the cfDNA extracted from draw 2 was insufficient for mutational analysis. Draw 1 analysis confirmed the *ERBB2* mutation (L755S) identified in the tumor pathology report prior to treatment. No additional mutations were detected.

**Fig. 6:**
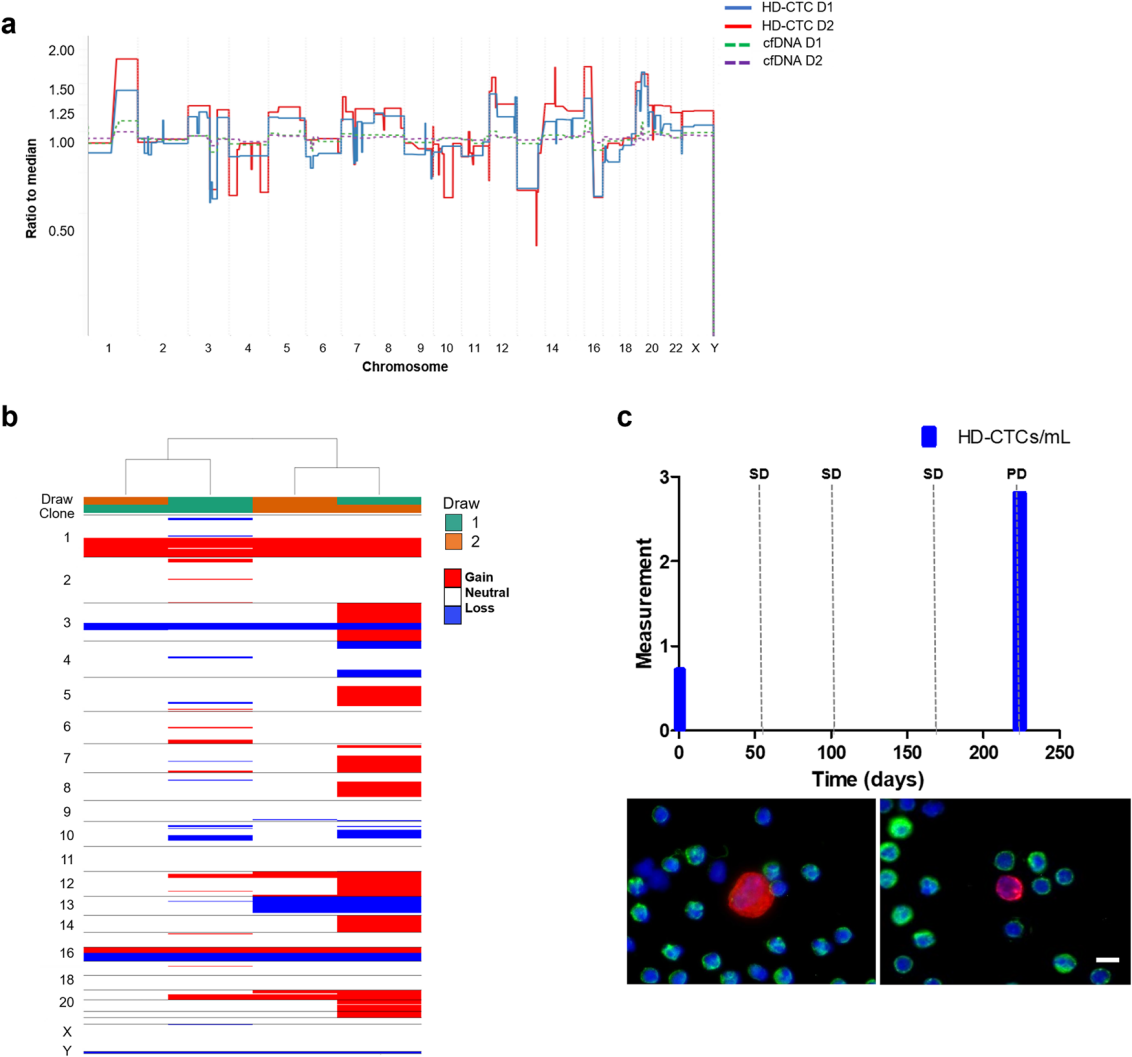
Comprehensive analysis of the liquid biopsy for Patient 5. **a** Similar clonal architecture identified in the CTCs was also detectable in the cfDNA, with a greater tumor fraction present in follow-up sampling. **b** Heatmap and phylogenic tree of single cell CNAs across the entire population of cells isolated. Draw number and clones are identified using color key. **c** Longitudinal analysis of CTCs in Patient 5. SD stable disease, PD progressive disease. Representative images of CTCs taken at 400X. Scale bar = 10 µm. White: ER, Red: CK, Green: CD45, Blue: DAPI.

Very few CTCs were detected in samples from Patient 5. Patient 5 had 0.76 CTCs/ml at draw 1 and stable kinetics to draw 2 with 2.81 CTCs/ml (Fig. 6c). At the time of draw 2 after 224 days since the start of therapy, this patient was determined to have progressive disease. The CTCs isolated from both draws shared a similar genomic architecture to the cfDNA (Fig. 6a, b), despite the identification of only a few CTCs per sample. The cells from draw 1 had a greater GI (mean = 606.13) than those from draw 2 (mean = 279.42). The relatively high GI observed in these draws may be indicative of poor prognosis as it suggests the presence of genomic alterations or large-scale state transitions that are maintained despite therapeutic intervention.

### Cohort overview of the liquid biopsy

Each PB sample was analyzed for the abundance of CTCs. The CTC enumeration per patient over the course of treatment with corresponding measurements of carcinoembryonic antigen (CEA), cancer antigens (CA27.29 and CA15-3), and/or disease status has been provided. Overall CTC enumeration alone was not sufficient to predict clinical response. Patient 3 was the only patient in this small cohort that had an overall increase in CTCs/ml from baseline to the last sample collected.

Morphological analysis of each CTC detected was conducted, with the nuclear area, nuclear eccentricity, and biomarker signal intensity presented as the SDOM (Supplemental Fig. 2). The morphology of the rare cells detected in the liquid biopsy were different between patients. Patient 3 had a morphologically distinct population of CTCs determined to be significantly different in all four morphometric parameters compared to CTCs from patients 1 and 2, presenting with a smaller, rounder nucleus, bright CK intensity, and minimal ER signal. The CTCs detected in Patient 4 were significantly lower in CK signal compared to Patients 3 and 5 but did not differ from Patients 1 and 2 in the other three variables analyzed. The CTCs detected in samples from Patient 5 were highly variable in nuclear area and eccentricity, with minimal ER signal intensity and a bright CK signal intensity compared to Patients 1 and 2. Further investigation of CTCs at the single cell level may provide insight into patient clinical response to treatment through analysis of the morphometric and genetic landscapes.

Genomic analysis of the single CTCs and bulk cfDNA was conducted to assess the genomic landscape of the cancer and its evolution during treatment with the hope of identifying potential genomic aberrations unique to disease progression or treatment resistance. The cfDNA GI score for Patient 3 was higher than that of any other patient. Interestingly, Patient 3 also presented with a new *ERBB2* mutation at draw 2, which was the same mutation detected in the tumor pathology of Patients 4 and 5. The increase in GI and gain of mutation during therapy, may be indicative of disease aggressiveness and lack of response to treatment.

**Fig. 7:**
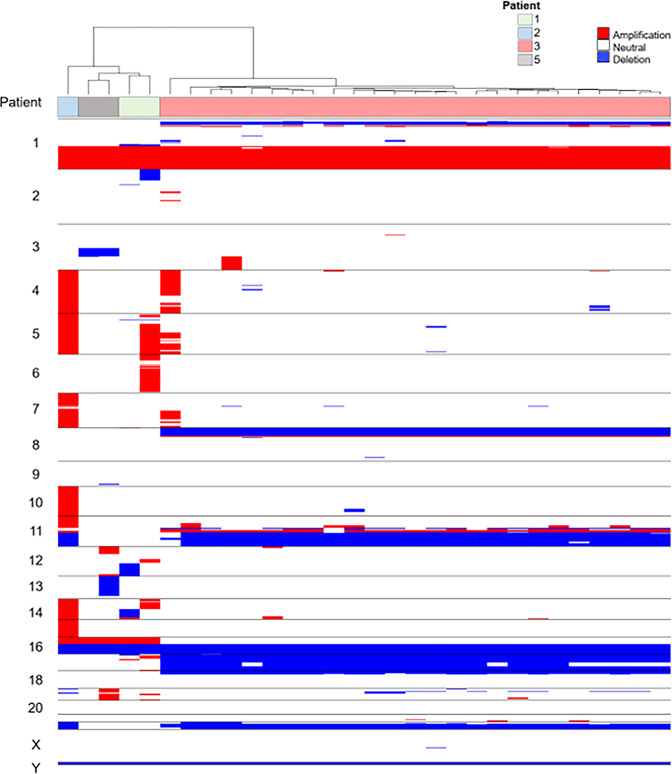
Genomic analysis of single cells isolated from the liquid biopsy from mBC patients receiving neratinib treatment prior to progression. Heatmap and phylogenic tree of CNVs across the entire population of cells from patient PB samples collected prior to progression. Patient number is identified using color key.

Hierarchical clustering of CNA profiles of CTCs isolated from the last draw collected prior to progression indicates that CTCs from Patients 1, 2, and 5 are more similar based on single cell genomics, than Patient 3. Additionally, we find common chromosomal 1q gains and 16p losses across cells from Patients 1, 2, 3, and 5 (Fig. 7). By aligning the CNA profiles to the COSMIC catalogue of somatic mutations in cancer we identified 30 genes that are similarly altered in CTCs prior to progression (Supplemental Table 2). Further exploration of these alterations may provide insight into treatment sensitivity or mutation driven resistance mechanisms. In luminal BC, the most frequent CNAs include whole arm gains of chromosomes 1q, 8q and 16p along with losses of 8p, 16q, 17p and 22q. Also common at metastasis are amplification of 11q13 and loss of the distal half of 11q. All four of the cases where a CNA profile was detected showed at least five of these early luminal characteristic CNAs, along with other less frequent events, indicating a luminal, as opposed to basal-like, origin.

## Discussion

Genetic heterogeneity between and within tumors is a major factor in determining cancer progression and therapy response. The use of a comprehensive liquid biopsy in the clinic may allow for the detection of dynamic tumor heterogeneity, stratification of patients onto targeted therapies, monitoring of response to treatment, and identification of acquired resistance on therapy. This is especially useful in an age of precision oncology. The importance of genomic sequencing is apparent with the identification of rare genetic subtypes of cancer, such as the patients presented here with *ERBB2* mutant, non-amplified mBC. The most commonly mutated region of *ERBB2* in all cancer types is the kinase domain (66%), followed by the extracellular domain (26%), and lastly the transmembrane/juxtamembrane domain (8%). These regional mutations were represented in Patients 2/4/5, Patient 1, and Patient 3, respectively, at enrollment. The specific location of mutations in HER2 could be a good biomarker for the efficacy of targeted HER2 therapy. We observed an average response to neratinib treatment for Patients 2, 4, and 5, all of whom harbored mutations in the tyrosine kinase domain, confirming sensitivity previously reported in other studies^13,45–48^. Additionally, Patient 1 presented with the S310F mutation and was found to be an excellent responder to neratinib, supporting the hypothesis that the S310F/Y mutation in the extracellular domain is sensitive to anti-HER2 therapy containing neratinib^49–51^. Contrary to the outcome observed for Patient 3, neratinib efficacy has been reported in a *ERBB2* I655V transmembrane domain mutation in different lung cancer cell lines^52^, suggesting the S653C mutation may be functionally different than the I655V mutation. Taken together this suggests the efficacy of neratinib is mutation specific and exploring the mechanism of action through characterization of the dynamic genomic landscape in each patient can advance our understanding of the HER2 genetics and its relationship with the efficacy of specific anti-HER2 treatments.

The liquid biopsy is a source of multiple analytes that could be used clinically to monitor treatment sensitivity and resistance^53^. The HDSCA workflow provides the opportunity to analyze both CTCs and cfDNA to characterize the heterogeneity associated with specific subsets of BC. The data presented here indicates a large degree of tumor heterogeneity in heavily treated tumors when characterized both morphometrically and genomically. Despite several differences in the cfDNA from the CTCs sequenced in this study, we want to highlight the fact that the CNA profiles share similarities suggesting that the CTC detected is indeed part of a clonal population contributing to the cfDNA, yet the higher resolution at the single cell level of the CTC provides for the ability to see specific differences. Although cfDNA analysis can provide valuable insight for targeted drug selection, it lacks information on protein expression as well as single-cell resolution to characterize genomic heterogeneity available from CTCs. Based on these observations it is clear that understanding the complexity of this disease over the time course of treatment, will require a comprehensive liquid biopsy characterization approach with the combined analysis of CTCs, cfDNA, and other blood-based analytes. Through the genomic sequencing of CTCs and cfDNA, we can monitor the evolution of resistance or lack of treatment efficacy, due to innate resistance, in real time, which may help clinicians exclude patients that will not benefit from this targeted treatment. While only limited alternatives might currently exist, it also enables the drug development community to more effectively initiate new discovery programs to target this type of disease.

Ma et al. demonstrated a clinical benefit rate for neratinib treatment of 31% in patients with HER2-mutated, non-amplified metastatic BC^54^. By utilizing retrospective ctDNA sequencing to detect HER2 mutations, they showed that patients with on-target effects had a decrease in HER2 mutant allele frequency while those patients that progressed had an increase in mutant allele frequency. In the study presented here, we present similar findings related to an increase in the cfDNA tumor fraction in a progressive disease patient. Additionally, a study by Hanker et al. used next generation sequencing of plasma to reveal *ERBB2* L869R and T798I mutant allele frequencies in a patient with acquired resistance to neratinib^55^, showing the utility of cfDNA in the identification of subclonal drug-resistant mutations upon clinical progression. By using the plasma as a repository for genomic alterations, we may monitor the overall tumor evolution in response to therapy. Beyond the cfDNA, unique to the study presented here is the corresponding genomic analysis of the single CTCs, while a regular tissue biopsy of a single lesion may not produce a complete profile of mutations, the liquid biopsy can provide both cellular and acellular information for bulk and single cell ctDNA comparison.

GI is characteristic of most cancers in which chromosome structure and number change over time in neoplastic cells compared to normal cells. The comprehensive liquid biopsy presents the opportunity to utilize CTC genomic events to follow progression of the disease and to infer some hints about actual mechanisms. For example, Patient 3 had a gain at chromosome 11q13 for the oncogene *CCND1* encoding cyclin D1, a cell cycle regulatory protein involved in mammary tissue development and carcinogenesis^56^. They also had genomic losses in a number of chromosomes characteristic of BC that were associated with poor prognosis, such as a loss of chromosome 8p, which is linked to an unfavorable tumor phenotype with advanced tumor stage and high tumor grade, rapid cellular proliferation, and poor clinical outcome^57–60^. Patient 3 also had observable alterations in the distal part of chromosome 11q containing a number of DNA repair genes, such as the ataxia telangiectasia mutated (*ATM*) and checkpoint kinase 1 (*CHK1)*, in which losses contribute to defective repair machinery. Additionally, the tumor suppressor gene *TP53* was compromised, which also affects DNA damage repair mechanisms. Taken together the genomic profile of the CTC clonal population from Patient 3 suggests a lack of cell cycle constraint and the reduced ability to repair DNA damage, likely contributing to the lack of clinical benefit seen with combinational neratinib and fulvestrant therapy. Interestingly, patients with concurrent aberrations in cell cycle checkpoints driven by *TP53* mutations have previously been associated with a lack of clinical benefit from neratinib^51^. This indicates that genomic analysis of the patients’ CTCs could identify those that would benefit from alternative treatment options.

Characterization of the comprehensive liquid biopsy allows us to understand the heterogeneity of the disease. Each compartment (cellular or acellular) and molecular analysis conducted (morphometric, CNA, or SNV) provides a unique analyte for distinct tumor characterization and insight into the personalized disease response to treatment. Single cell analysis provides information on the morphological and genomic tumor heterogeneity, while providing insight into resistance mechanisms and potential new targets for drug development. Analyzing cfDNA provides information on the bulk genomic profile, showing the most prominent genomic profile present. The results suggest that using a combination of SNVs and CNAs to interrogate the liquid biopsy is necessary to understand the overall mutational burden within each patient as well as the patient phenotypes across the cohort. CNAs may show the heterogeneity of the genomic landscape or loss of heterozygosity through identification of a single or multiple clonal populations, while also determining if they co-occur with SNVs or indels. The ability to characterize tumor heterogeneity using a single platform with comprehensive single cell DNA and cfDNA analysis to co-detect both SNVs and CNAs is highly impactful.

The main limitation to this study is the lack of corresponding data from the solid biopsy for comparison to the liquid biopsy data. Multiple biopsies are frequently needed to characterize underlying genomic alterations associated with treatment resistance in cancer. However, the invasive nature of tumor-tissue biopsies makes utility of the liquid biopsy specimens an attractive alternative to multiple tissue biopsies. The level of concordance between sequencing results obtained using tumor tissue and the liquid biopsy still remains in question. In future trial designs, we will incorporate access to any available solid tissue for parallel analysis. Additionally, more frequent sample collections for all patients would allow for a more in-depth longitudinal analysis of the evolution of disease. As an example, Patient 5 only had 2 samples collected and analyzed 224 days apart. If additional samples had been collected during this period, further spatial and temporal information on the disease, such as clonal evolution, could be utilized to understand the patient response in real time during the course of therapy.

The data presented in this small cohort study demonstrates the feasibility of real-time molecular profiling of the liquid biopsy using the HDSCA workflow, while proposing the use of morphometric and genomic analysis as a prognostic tool to advance personalized oncology. Further analysis is warranted to provide additional insight into the prognostic significance of CTC morphology, CTC clonality or single cell mutations, and cfDNA GI to predict the response of individual patients to targeted therapies. Identifying genomic alterations associated with clinical response and subsequent progression after targeted therapy demonstrates the timeframe of tumor evolution in response to therapy. The comprehensive liquid biopsy as implemented in the HDSCA workflow provides a framework to quantify and monitor disease progression to facilitate delivery of personalized medicine for BC.

## Methods

### Patient cohort and sample collection

Here we evaluated the clinical response and genomic profiles of five post-menopausal patients with metastatic *ERBB2* mutant, non-amplified BC from the MutHER (NCT01670877) or SUMMIT (NCT01953926) trials being seen at the USC Norris Comprehensive Cancer Center. Patients on this study had an average of 5.4 lines of therapy before being placed on a combined treatment consisting of neratinib and fulvestrant. Eligibility required identification of somatic *ERBB2* mutation with the absence of HER2 expression or *ERBB2* amplification (0 or 1+ by immunohistochemistry or non-amplified by FISH). Patients received neratinib 240 mg PO daily (28-day cycle) and 500 mg fulvestrant (28-day cycle) with prophylactic loperamide to reduce the frequency of diarrhea. Treatment was continued unless there was occurrence of disease recurrence, intolerable adverse events, or consent withdrawal. Dose reduction of neratinib was allowed in cases of severe toxicity, with treatment cessation allowed if lowest dose was not tolerated or treatment was interrupted for more than three weeks. Dose reductions were mandated for grade 3 diarrhea. During the treatment period, PB samples were collected at multiple timepoints as a liquid biopsy to molecularly characterize CTCs and cfDNA. The methods were performed in accordance with relevant guidelines and regulations and was approved by the institutional review board of the University of Southern California’s Keck School of Medicine (Los Angeles, California, United States). Patients recruited in these studies provided informed written consent. These patients had a potential for up to 14 study related PB draws at intervals of 8–12 weeks, coordinated with routine clinic visits. This collection scheme was designed with a primary goal of screening the liquid biopsy and the reproducible identification of CTCs, with a secondary goal being for clinical correlations.

### Characterization of cfDNA from liquid biopsies

#### Isolation of plasma and cfDNA

The cfDNA was isolated from plasma samples from BC patients. Plasma was isolated by centrifuging the PB samples at 2,000 g for 10 min, allowing for collection of at least 2 ml per blood tube. The plasma was then transferred to a new tube and centrifuged at 14,000 g for 10 min.

Purification of cfDNA from ≥2 ml of frozen plasma samples was conducted using the QIAamp Circulating Nucleic Acid Kit (QIAGEN, Cat# 55114) following the manufacturer’s protocol. If available plasma was more than 5 ml, protocol was performed in duplicate. Eluted cfDNA concentration was quantified by Qubit (Thermo Fisher).

#### Copy number alteration (CNA) analysis of cfDNA

Five nanograms of cfDNA was used for library construction using the NEBNext Ultra™ II DNA Library Prep Kit for Illumina (New England Biolabs, Cat#. E7645L) according to manufacturer’s protocol. After adaptor ligation, a clean-up without size selection of the adaptor-ligated DNA was performed using AMPure Beads (Beckman Coulter Inc., Cat# A63882) followed by seven cycles of PCR to generate a library of sufficient yield. The concentration of the constructed library DNA was quantified by Qubit high-sensitivity dsDNA assay (Thermo Fisher), while the distribution of library size was assessed using the Agilent 2100 Bioanalyzer (High-Sensitivity DNA Assay and Kit, Agilent Technologies, Cat#. 5067-4626).

The individual libraries from each plasma sample were pooled at equimolar concentrations. The pooled libraries were cleaned using AMPure XP Beads and sequenced using the Illumina NextSeq 500 or the HiSeq2500 SR50. Resulting fastq files were aligned to the hg19 reference genome using the Bowtie algorithm, while the BAM files were sorted, and PCR duplicates were removed using SAMtools. A Python script was used to count the number of reads in each bin^61,62^. An R script utilizing the Bioconductor package DNAcopy_1.26.0 (http://bioconductor.org/packages/DNAcopy/) normalized and segmented the bin counts per sample into a CNA profile.

Analysis of cfDNA includes detecting both healthy cfDNA in the PB as well as tumor-derived DNA (ctDNA) from various tumor cells. Tumor fractions were estimated using ichorCNA^63^ with a bin size of 1 million base pairs (bp). We have confidence in the tumor fraction detected with the ichorCNA method down to 10%. Alternatively, the read depth behind each alteration in cfDNA is theoretically proportional to the reads mapping to the reference CNA from the same biopsy. Therefore, the HDSCA percent ctDNA detected in cfDNA was conducted by using CTCs from the same sample as a reference CNA. We calculated the ratio of each cfDNA variant to each reference CTC variant by overlaying the CNAs at the appropriate ratio, to visually compare and confirm the profile of frequencies. We have confidence in the detection of ctDNA in cfDNA with this method down to 5%.

#### Single nucleotide variation (SNV) analysis of cfDNA

Samples with a minimum of 15 ng of cfDNA extracted from the plasma were candidates for mutational analysis, with the goal of analyzing a minimum of two timepoints per patient (Table 2; Supplemental Table 4). The cfDNA of all samples which met the minimum starting material requirements (*n* = 12), was analyzed for SNVs using the Ion Torrent Oncomine Pan-Cancer Cell-Free Assay (ThermoFisher, Cat# A37664) or the Breast cfDNA Assay (ThermoFisher, Cat# A31183) in combination with the Ion Torrent Chef system and S5XL sequencer (ThermoFisher, Waltham, MA) according to the manufacturer’s instructions. The Pan-Cancer Cell-Free Assay is designed to sequence over 900 hotspots of 52 cancer genes, 12 copy number genes, and the full length of the *TP53* gene. The Breast cfDNA Assay enables analysis of 10 genes frequently mutated in BC with over 150 hotspots covered. Samples from Patient 3 were analyzed by the Breast cfDNA Assay while all other samples were analyzed by the Pan-Cancer Cell-Free Assay. Fragment size of constructed libraries were tested using the Agilent 2100 Bioanalyzer (High-Sensitivity DNA Assay and Kit, Agilent Technologies, Cat# 5067-4626). Selection of samples was independent of the estimated tumor fraction. Data was processed with OncoMine Pan-Cancer or Breast Liquid Biopsy workflow on Ion Reporter.

### Identification and characterization of CTCs

PB was collected in a preservative blood collection tube (Cell-Free DNA BCT, Streck, Omaha, NE) at multiple timepoints during treatment. CTCs were identified using the HDSCA workflow. Overall performance, including sensitivity and specificity, in both healthy donors and cancer patients was described previously^34–36^. Briefly, following plasma extraction and red blood cell lysis a monolayer of all nucleated cells was attached to custom-made glass slides (Marienfeld-Superior, Germany).

Slides were stained using an IntelliPATH FLX™ autostainer (Biocare Medical LLC, Irvine, CA, USA) in batches of 50. All steps were performed at room temperature. Slides were fixed with 2% neutral buffered formalin solution (VWR, San Dimas, CA) for 20 min. Non-specific binding sites were blocked with 10% goat serum (Millipore, Billerica, MA) for 20 min. Slides were incubated with Alexa Fluor 647 conjugated anti-CD45 (1:125; clone: F10-89-4, MCA87A647, AbD Serotec, Raleigh NC, USA), anti-ER (1:250, clone SP1, MA5-14501, ThermoFisher, Waltham, MA, USA) followed by an Alexa Fluor 488 secondary (1:1000, A-11008, Invitrogen, Waltham, MA), and antibodies against cytokeratins (panCK, 1:100, clones: C-11, PCK-26, CY-90, KS-1A3, M20, A53-B/A2,C2562, Sigma, St. Louis MO USA; CK19, 1:100, clone: RCK108, GA61561-2, Dako, Carpinteria, USA) followed by an Alexa Fluor 555 secondary (1:500, A21127, Invitrogen, Waltham, MA). DAPI was used to counterstain nuclei. Candidate CTCs were located and identified by computational analysis of imaging data obtained from an automated digital microscopy technique (100X magnification). A hematopathologist-trained technical analyst subsequently analyzed and interpreted the images of CTC candidates. Cells were classified as CTCs using the following criteria: CD45 negative, cytokeratin (CK) positive, morphologically distinct from surrounding white blood cells (WBCs), with an intact nucleus, without observable apoptotic changes or a disrupted appearance. WBC counts of the whole blood were measured (Medonic M-series Hematology Analyzer, Clinical Diagnostic Solutions Inc., Fort Lauderdale, FL, USA) and the number of cells detected per slide by the assay were used to calculate the actual volume of whole blood that was analyzed per slide. All cells were counted individually, however, results are presented as fractional values of CTCs/ml. In the case of CTC clusters, defined by two or more cells, each individual cell in the cluster was counted, rather than reporting a single entity. Sample positivity was defined as ≥1 CTC/ml. The CTC kinetics between sample dates in individual patients were observed to follow three distinct patterns while on therapy: an increasing, decreasing, or unchanged (stable) CTC count. Stability was defined as <5 CTC/ml change from the previous draw.

In addition to CTC enumeration, the high-content data consisted of additional parameters: relative nuclear area and eccentricity per CTC, as well as relative CK and ER staining intensities per CTC. Fluorescent channel signal intensity is presented as the Standard Deviation Over the Mean (SDOM) signal intensity of the cell of interest based on the background signal of the nearest 50 WBCs in the frame.

For each autostainer run, the variability was assessed using a previously established quality assurance and quality control program which requires performance of spike-in experiments with MCF-7, MDA-MB-231, and SKBR3 cells in normal control PB samples^36,37,39^. A minimum of two positive control slides were routinely included in each autostainer run along with slides from BC patients’ PB samples and were subsequently scanned and evaluated.

### Single cell genomic analysis

#### Isolation of single cells

First, CTCs were relocated on the glass slide and re-imaged at 400X magnification for detailed morphometric analysis. Then, an Eppendorf Transfer Man NK2 micromanipulator was used to dislodge and capture the cell of interest from the slide into a 25° jagged micropipette by applying fluid suction. The cell was then deposited inside a 0.2 ml PCR tube containing 1 µl of Tris-EDTA buffer. Samples were immediately frozen and stored at −80 °C until subjected to whole genome amplification (WGA) and sequencing library construction.

#### WGA of single cells

WGA of single cells was conducted using the WGA4 Genomeplex Single Cell Whole Genome Amplification Kit (Sigma Aldrich Cat#. WGA4). Amplification was carried out according to the manufacturer’s protocol, with the exception of amplification performed with 23 PCR cycles. In short, the cells were lysed by adding 1.5 µl lysis buffer (1:1 solution of 100 mM DTT + 400 mM KOH) to each tube containing a single thawed cell and subsequently incubated for 2 min at 95 °C. A master mix containing 6.5 µl 10 mM Tris-HCl-EDTA pH 8.0 per reaction and 1 µl of the 10x Single Cell Lysis & Fragmentation Buffer was added to the cold reaction. The samples were incubated for 4 min at 99 °C. Successful amplification was confirmed by gel electrophoresis (1.5% agarose gel) with a DNA smear around 200–1200 bp. Further, DNA was purified using a QIAquick PCR Purification Kit (Thermo Fisher, Cat#. K210012 with elution in 50 µl of TE-buffer. Concentration of purified DNA was quantified with Qubit Fluorometric Quantification (Thermo Fisher). Amplified DNA was sheared using sonication (Covaris S2/E210 focused-Ultrasonicater) with the microtube setup and the 200 bp target size protocol for DNA shearing. Final sample volume was 75 µL.

#### CNA analysis of single cells

CNA analysis utilized 50 ng of amplified and sonicated DNA from single cells for library construction using the NEBNext Ultra DNA Library Preparation Kit from Illumina (New England Biolabs, Cat#. E7370L). The library concentration was quantified with Qubit (Thermo Fisher) and the expected library size distribution of 300–500 bp was confirmed using the Agilent 2100 Bioanalyzer (High-Sensitivity DNA Assay and Kit, Agilent Technologies, Cat#. 5067-4626). The individual libraries from single cells with identifiable and distinguishable indices were pooled at equimolar concentration (approximately 6 ng per library; 50–70 single cells) and cleaned using AMPure XP Beads (Beckman Coulter Inc., Cat# A63882).

Libraries were sequenced using the Illumina NextSeq 500 or the HiSeq2500 SR50. 30 bp were trimmed off the ’5 end of each read to remove the WGA4 adapter sequence before alignment to the hg19 reference genome using the Bowtie algorithm. The resulting BAM file was sorted, and PCR duplicates were removed using SAMtools. The number of reads falling into each of 5000 ‘bins’ comprising the UCSC reference genome, was calculated using a previously published Python script [9]. Finally, an R script utilizing the Bioconductor package, DNAcopy_1.26.0 (http://bioconductor.org/packages/DNAcopy/), was used to normalize and segment the bin counts across each chromosome generating a genome-wide CNA profile^64,65^. We defined clonality as two or more cells that share two or more genomic alterations denoting replicating neoplastic cells.

Multiple CNA profiles are presented in the form of a heatmap to provide a visual representation of the CNA data. Each individual CTC or cfDNA profile is represented as a column, while the rows indicated the chromosomal regions along the genome. For each sample, the segments are represented by a rectangle plotted in a color corresponding to the difference between the segment copy number value and the limits (upper 1.25, lower 0.75). The magnitude of the estimated copy numbers is presented in three levels: neutral in white, gain in red, and loss in blue. The timepoint of collection is indicated by Draw number and each unique clone identified through a ward.D methodology which is color coded for clarity.

The genomic instability (GI) was calculated as the ratio to the median (RM) using a hyperbolic tangent function. We modified the distribution from Balakrishnan to meet the logistic nature of the CNA profile^66^. Utilizing the logarithmic function allows for the score of one complete loss to be equivalent to the score of one complete gain. The further the RM is from 1, the higher the score. Therefore, the higher the GI score, the more aberrant the CNA profile. A CNA neutral profile has an GI score ≤30, while a CTC which carries a few chromosomal breaks typically has an GI score >100. The calculation of GI is a summation for every bin of the following:

$$\tanh \left( {3 \times \left| {\log \left( {\frac{x}{{median\left( x \right)}}} \right)} \right| - 2} \right) + {{{\mathrm{tanh}}}}(2)$$

### Statistical analysis

Statistical two-sided analyses were performed using R (R-4.0.3., Boston, MA). Groups were compared using the Kruskal–Wallis Test, Welch’s *t*-test, or Wilcoxon signed rank test, as appropriate. Correlation was calculated using the Spearman’s rank-order correlation test or Chi Square test. Statistical significance was determined at a *p* value of ≤0.05.

### Reporting summary

Further information on research design is available in the Nature Research Reporting Summary linked to this article.

## Supplementary information

Supplemental Material

Reporting Summary Checklist

## Data Availability

All data discussed in this manuscript are either included in the main manuscript text or in the Supplementary Information Files. Some of the data can be accessed through our website http://pivot.usc.edu/. The sequencing data of the single cells and cfDNA is available through the BloodPAC Data Commons Accession ID “BPDC000119”.

## Code availability

The code used to analyze the single-cell and cfDNA genomics data uses standard third-party open-source libraries and packages in R and Python. The code used to identify CTCs in the HDSCA workflow uses custom code and is proprietary and licensed to Epic Sciences for commercial use.
