# Supplementary material for: Disease characterization in liquid biopsy from HER2-mutated, non-amplified metastatic breast cancer patients treated with neratinib
Source: NPJ Breast Cancer. 2022 Feb 18;8:22. doi: 10.1038/s41523-022-00390-5 (PMC8857263; doi:10.1038/s41523-022-00390-5)
Supplement: Supplementary file 1 — Supplemental Material [file 41523_2022_390_MOESM1_ESM.pdf]

Supplemental Discussion

By design, the HDSCA workflow uses an enrichment-free, direct imaging analysis approach to identify rare cells and characterize them at the cellular, protein and molecular level, developed at The Scripps Research Institute and licensed to Epic Sciences for commercial development [1-3]. The HDSCA workflow maintains a complete chain of custody from the patient data to the bulk sample to the individual analyte. The rare cell identification part of the workflow is extensively validated in both the RUO and CLIA certified settings. Prior studies have shown a concordance between HD-CTCs and the solid tumor [4,5]. We have also used single-cell sequencing and targeted multiplexed proteomic analysis to characterize rare and common cells [5-11]. Performance of the HDSCA workflow in healthy donors, as well as the sensitivity and accuracy of this assay was described previously [12]. Briefly assay linearity and sensitivity was tested using various numbers of breast cancer cell line SKBR3 cells spiked into normal control blood and processed according to the HDSCA workflow. The correlation coefficient of expected to observed SKBR3 cells was 0.9997. In this study ≥ 5 HD-CTCs/mL were found in 70% of the breast cancer patients (n=30, mean = 56.8) and 0% of normal controls (n=15, mean = 0.6).

Our previous validation studies have compared the differential performance of five blood collection tubes (BCTs) and four time points from blood draw to assay (TTA) across the HDSCA workflow from cell enumeration to the downstream evaluation of single-cell genomics using standard operating procedures (SOPs) [10,13]. We determined the optimal BCT was the Streck Cell-free DNA tube and performance is optimized for high detection levels of rare tumor cells when blood is processed within 48 hours of collection. A Quality Management Plan containing QA/QC SOPs was established with a scientific advisory team which included representatives from the NCI, the College of American Pathologists (CAP), and various academic institutions and are being implemented as part of BloodPAC Consortium protocols [14,15].

Supplementary Figures and Tables

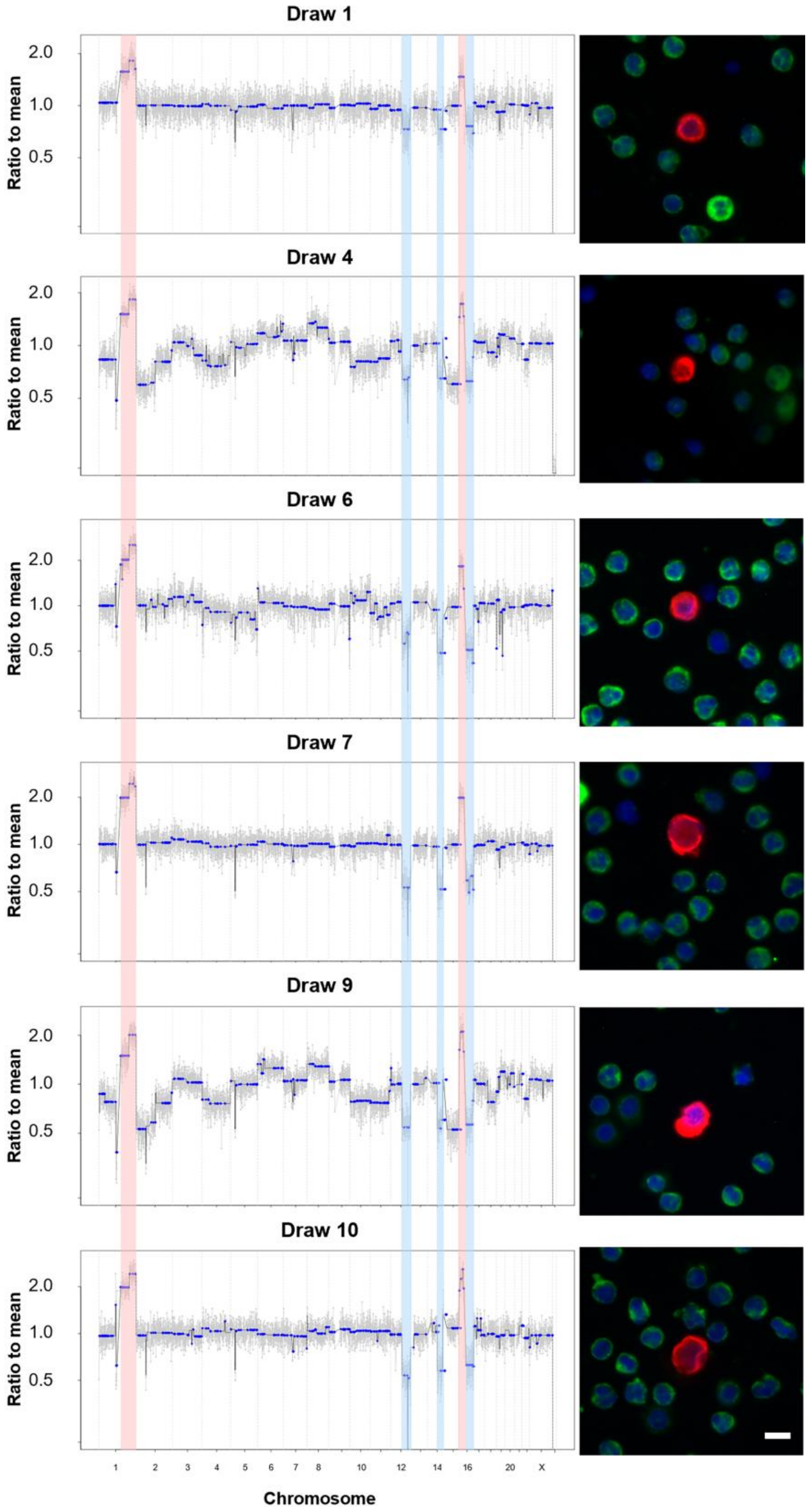

Supplementary Figure 1. Cell lineage of dominant clone starting from Patient 1 draw 1. CNV profile and matching micrographs for CTCs detected in the liquid biopsy from Patient 1. Main alterations that define this clonal population are highlighted with gains in red and losses in blue. Through this analysis, the lineage of the cancer cells can be tracked from draw to draw. Images taken at 400X. Scale bar = 10µm. DAPI is in blue, CK in red, ER in white, and CD45 in green.

Supplementary Table 1. Genes that are similarly altered from Patients 1, 2, 3, and 5. The CTCs isolated from the draws taken prior to progression indicated patients share a gain in chromosome 1q (red) and a loss in chromosome 16q (blue).

Symbol	Name
PDE4DIP	Phosphodiesterase 4D interaction protein (myomegalin)
BCL9	B-cell CLL/lymphoma 9
AF1Q	ALL1-fused gene from chromosome 1q
ARNT	Aryl hydrocarbon receptor nuclear translocator
TPM3	Tropomyosin 3
MUC1	Mucin 1, transmembrane
NTRK1	Neurotrophic tyrosine kinase, receptor, type 1
PRCC	Papillary renal cell carcinoma (translocation-associated)
IRTA1	Immunoglobulin superfamily receptor translocation associated 1
SDHC	Succinate dehydrogenase complex, subunit C, integral membrane protein, 15kDa
FCGR2B	Fc fragment of IgG, low affinity IIb, receptor for (CD32)
PBX1	Pre-B-cell leukemia transcription factor 1
PMX1	Paired mesoderm homeo box 1
ABL2	V-abl Abelson murine leukemia viral oncogene homolog 2
TPR	Translocated promoter region
CDC73	Cell division cycle 73
PTPRC	Protein tyrosine phosphatase, receptor type, C
MDM4	Mdm4 p53 binding protein homolog
ELK4	ELK4, ETS-domain protein (SRF accessory protein 1)
SLC45A3	Solute carrier family 45, member 3
H3F3A	H3 histone, family 3A
HIST1H3B	Histone cluster 1, H3b
FH	Fumarate hydratase
CYLD	Familial cylindromatosis gene
HERPUD1	Homocysteine-inducible, endoplasmic reticulum stress-inducible, ubiquitin-like domain member 1
CDH11	Cadherin 11, type 2, OB-cadherin (osteoblast)
CBFB	Core-binding factor, beta subunit
CDH1	Cadherin 1, type 1, E-cadherin (epithelial) (ECAD)
MAF	V-maf musculoaponeurotic fibrosarcoma oncogene homolog
CBFA2T3	Core-binding factor, runt domain, alpha subunit 2; translocated to, 3 (MTG-16)

Supplementary Table 2. CfDNA isolated form the liquid biopsy. DNA was extracted from 2 – 4 mL plasma collected from the peripheral blood. The detection limit of ICOR ctDNA is 10%, the detection limit of CSI-Cancer ctDNA is 5%. Samples below this are denoted as <10 or <5, respectively.

Patient	Draw	ctDNA%	Genomic Instability Score
1	1	<5	15.50
	2	<5	11.80
	3	<5	10.20
	4	<5	14.00
	5	<5	13.60
	6	<5	15.50
	7	5.88	13.00
	8	<5	11.00
	9	10.00	18.10
	10	16.67	26.00
2	1	<5	11.06
	2	<5	6.70
	3	<5	8.40
	4	<5	18.60
	5	7.46	27.00
	6	11.11	33.25
3	1	6.90	16.46
	2	62.50	172.80
4	1	<5	23.94
	2	<5	21.80
	3	<5	13.70
	4	<5	8.39
5	1	14.29	46.28
	2	9.62	15.88

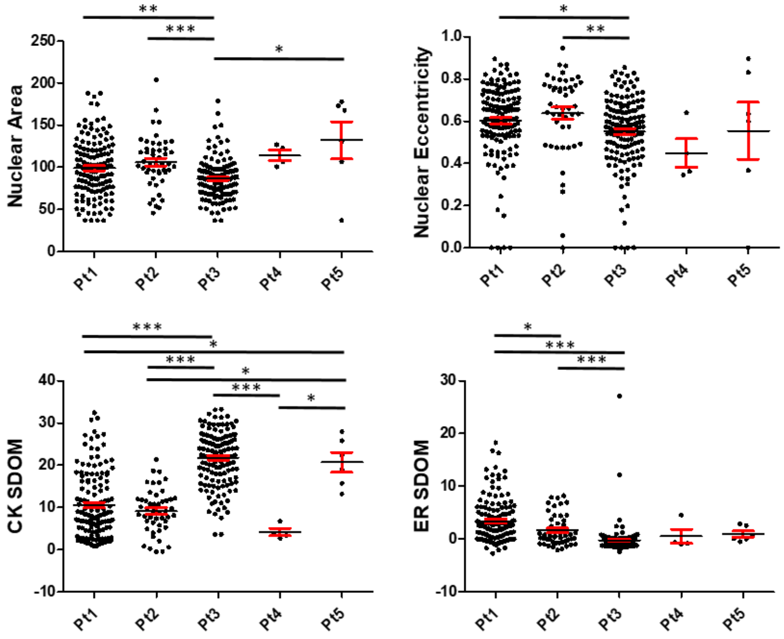

Supplementary Figure 2. Morphological analysis of CTCs detected in the liquid biopsy from metastatic breast cancer patients receiving neratinib and fulvestrant treatment. Statistical analysis of CTC morphometric parameters compared between patients. Mean and standard error of the mean are indicated in red. Significant difference * p-value ≤0.05, ** p-value ≤0.01, *** p-value ≤0.001.

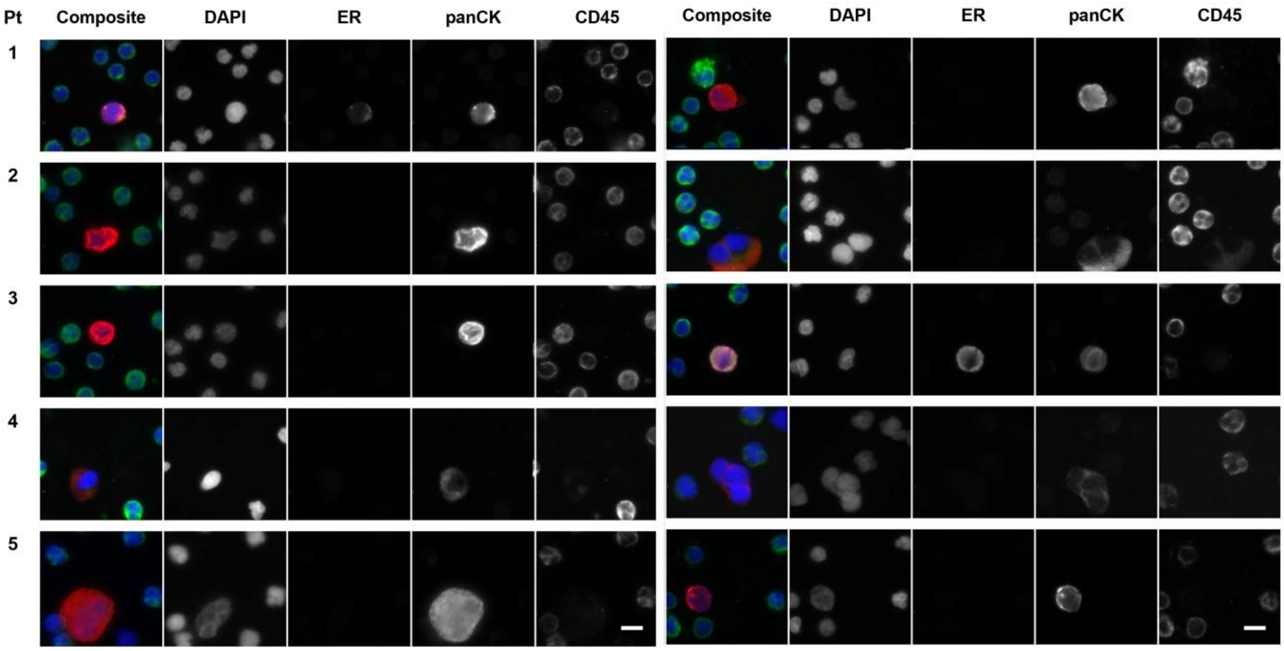

Supplementary Figure 3. Gallery of representative images of CTCs detected in each patient. The corresponding multichannel composite is shown in the left column. Images taken at 400X. Scale bar = 10μm. White: ER, Red: pan-cytokeratin (panCK), Green: CD45, Blue: DAPI.

Supplementary Table 3. Sequencing statistics for CfDNA SNV analysis by the Oncomine assay.

Pt	Draw	Locus	VAF%	Genes	AA change	Genotype	Raw Read Depth	Mol. Depth (unique reads)	WT Mol. Counts	Alt Allel Mol. Counts
1	4	chr17:7578212	0.0788	TP53	p.R213*	G/A	35453	2537	2533	2
1	8	chr17:7578212	0.4688	TP53	p.R213*	G/A	79255	8531	8491	40
		chr17:37880257	0.1252	ERBB2	p.I767M	C/G	62392	6387	6379	8
1	9	chr17:7578212	2.9102	TP53	p.R213*	G/A	39435	3230	3136	94
		chr17:37868208	0.1778	ERBB2	p.S310F	C/T	50980	3937	3930	7
1	10	chr6:152419923	0.0657	ESR1	p.Y537F	A/T	62560	7599	7594	5
		chr17:7578212	4.5476	TP53	p.R213*	G/A	72570	6355	6066	289
		chr17:37868208	0.2907	ERBB2	p.S310F	C/T	88325	6879	6859	20
		chr17:37880261	0.1223	ERBB2	p.D769H	G/C	70513	4905	4899	6
2	4	Not detected								
2	6	chr17:37880997	0.122	ERBB2	p.G776S	G/A	52300	4096	4091	5
		chr17:37880998	1.9387	ERBB2	p.G776V	G/T	52300	4178	4097	81
3	1	chr3:178936091	2.7324	PIK3CA	p.E545K	G/A	20646	2818	2741	77
		chr6:152332832	0.5038	ESR1	p.E380Q	G/C	21604	2779	2765	14
		chr6:152419922	0.3323	ESR1	p.Y537N	T/A	16413	2708	2699	9
		chr17:7577538	0.3053	TP53	p.R248Q	C/T	16218	2293	2286	7
3	2	chr3:178936091	30.0664	PIK3CA	p.E545K	G/A	31860	5571	3896	1675
		chr3:178938934	0.0947	PIK3CA	p.E726K	G/A	31048	5280	5275	5
		chr6:152332832	0.0694	ESR1	p.E380Q	G/C	35430	5761	5757	4
		chr17:7577538	0.189	TP53	p.R248Q	C/T	14998	2645	2640	5
		chr17:7577547	0.1134	TP53	p.G245D	C/T	14998	2645	2642	3
		chr17:37880220	0.1305	ERBB2	p.L755S	T/C	11244	2298	2295	3
4	1	chr3:178952085	1.0284	PIK3CA	p.H1047R	A/G	47063	2528	2502	26
		chr17:7573933	0.3783	TP53	p.H365fs	T/TC	69317	3700	3686	14
		chr17:37880220	0.7893	ERBB2	p.L755S	T/C	47579	2407	2388	19
		chr17:37881000	0.9275	ERBB2	p.V777L	G/T	65758	3989	3952	37
4	2	Not detected								
4	4	chr3:178952085	0.5809	PIK3CA	p.H1047R	A/G	45363	6197	6161	36
		chr17:7577539	0.0776	TP53	p.R248W	G/A	71440	6438	6433	5
		chr17:37880220	0.784	ERBB2	p.L755S	T/C	45526	5229	5188	41
		chr17:37881000	0.4609	ERBB2	p.V777L	G/T	81367	9762	9717	45
5	1	chr17:37880220	14.3863	ERBB2	p.L755S	T/C	67444	8522	7296	1226

Supplementary References

1 Armstrong, A. J. *et al.* Prospective Multicenter Validation of Androgen Receptor Splice Variant 7 and Hormone Therapy Resistance in High-Risk Castration-Resistant Prostate Cancer: The PROPHECY Study. *J Clin Oncol* **37**, 1120-1129, doi:10.1200/JCO.18.01731 (2019).

2 Scher, H. I. *et al.* Assessment of the Validity of Nuclear-Localized Androgen Receptor Splice Variant 7 in Circulating Tumor Cells as a Predictive Biomarker for Castration-Resistant Prostate Cancer. *JAMA Oncol* **4**, 1179-1186, doi:10.1001/jamaoncol.2018.1621 (2018).

3 Scher, H. I. *et al.* Association of AR-V7 on Circulating Tumor Cells as a Treatment-Specific Biomarker With Outcomes and Survival in Castration-Resistant Prostate Cancer. *JAMA Oncol* **2**, 1441-1449, doi:10.1001/jamaoncol.2016.1828 (2016).

4 Gerdtsson, A. S. *et al.* Single cell correlation analysis of liquid and solid biopsies in metastatic colorectal cancer. *Oncotarget* **10**, 7016-7030, doi:10.18632/oncotarget.27271 (2019).

5 Malihi, P. D. *et al.* Clonal diversity revealed by morphoproteomic and copy number profiles of single prostate cancer cells at diagnosis. *Converg Sci Phys Oncol* **4**, doi:10.1088/2057-1739/aaa00b (2018).

6 Dago, A. E. *et al.* Rapid phenotypic and genomic change in response to therapeutic pressure in prostate cancer inferred by high content analysis of single circulating tumor cells. *PLoS One* **9**, e101777, doi:10.1371/journal.pone.0101777 (2014).

7 Gerdtsson, E. *et al.* Multiplex protein detection on circulating tumor cells from liquid biopsies using imaging mass cytometry. *Converg Sci Phys Oncol* **4**, doi:10.1088/2057-1739/aaa013 (2018).

8 Malihi, P. D. *et al.* Single-Cell Circulating Tumor Cell Analysis Reveals Genomic Instability as a Distinctive Feature of Aggressive Prostate Cancer. *Clin Cancer Res* **26**, 4143-4153, doi:10.1158/1078-0432.CCR-19-4100 (2020).

9 Ruiz, C. *et al.* Limited genomic heterogeneity of circulating melanoma cells in advanced stage patients. *Phys Biol* **12**, 016008, doi:10.1088/1478-3975/12/1/016008 (2015).

10 Shishido, S. N. *et al.* Preanalytical Variables for the Genomic Assessment of the Cellular and Acellular Fractions of the Liquid Biopsy in a Cohort of Breast Cancer Patients. *J Mol Diagn* **22**, 319-337, doi:10.1016/j.jmoldx.2019.11.006 (2020).

11 Thiele, J. A., Pitule, P., Hicks, J. & Kuhn, P. Single-Cell Analysis of Circulating Tumor Cells. *Methods Mol Biol* **1908**, 243-264, doi:10.1007/978-1-4939-9004-7_17 (2019).

12 Marrinucci, D. *et al.* Fluid biopsy in patients with metastatic prostate, pancreatic and breast cancers. *Phys Biol* **9**, 016003, doi:10.1088/1478-3975/9/1/016003 (2012).

13 Rodriguez-Lee, M. *et al.* Effect of Blood Collection Tube Type and Time to Processing on the Enumeration and High-Content Characterization of Circulating Tumor Cells Using the High-Definition Single-Cell Assay. *Arch Pathol Lab Med* **142**, 198-207, doi:10.5858/arpa.2016-0483-OA (2018).

14 Grossman, R. L. *et al.* Collaborating to Compete: Blood Profiling Atlas in Cancer (BloodPAC) Consortium. *Clin Pharmacol Ther* **101**, 589-592, doi:10.1002/cpt.666 (2017).

15 Grossman, R. L. *et al.* BloodPAC Data Commons for Liquid Biopsy Data. *JCO Clin Cancer Inform* **5**, 479-486, doi:10.1200/CCI.20.00179 (2021).
