# Supplementary material for: Disease characterization in liquid biopsy from HER2-mutated, non-amplified metastatic breast cancer patients treated with neratinib
Source: NPJ Breast Cancer. 2022 Feb 18;8:22. doi: 10.1038/s41523-022-00390-5 (PMC8857263; doi:10.1038/s41523-022-00390-5)
Supplement: Supplementary file 2 — Reporting Summary Checklist [file 41523_2022_390_MOESM2_ESM.pdf]

nature portfolio

Corresponding author(s): Peter Kuhn

Last updated by author(s): 2022/01/04

Reporting Summary

Nature Portfolio wishes to improve the reproducibility of the work that we publish. This form provides structure for consistency and transparency in reporting. For further information on Nature Portfolio policies, see our Editorial Policies and the Editorial Policy Checklist.

Statistics

For all statistical analyses, confirm that the following items are present in the figure legend, table legend, main text, or Methods section.

n/a	Confirmed	
☐	☒	The exact sample size (*n*) for each experimental group/condition, given as a discrete number and unit of measurement
☐	☒	A statement on whether measurements were taken from distinct samples or whether the same sample was measured repeatedly
☐	☒	The statistical test(s) used AND whether they are one- or two-sided<br>*Only common tests should be described solely by name; describe more complex techniques in the Methods section.*
☐	☒	A description of all covariates tested
☐	☒	A description of any assumptions or corrections, such as tests of normality and adjustment for multiple comparisons
☐	☒	A full description of the statistical parameters including central tendency (e.g. means) or other basic estimates (e.g. regression coefficient) AND variation (e.g. standard deviation) or associated estimates of uncertainty (e.g. confidence intervals)
☒	☐	For null hypothesis testing, the test statistic (e.g. *F*, *t*, *r*) with confidence intervals, effect sizes, degrees of freedom and *P* value noted<br>*Give P values as exact values whenever suitable.*
☒	☐	For Bayesian analysis, information on the choice of priors and Markov chain Monte Carlo settings
☐	☒	For hierarchical and complex designs, identification of the appropriate level for tests and full reporting of outcomes
☒	☐	Estimates of effect sizes (e.g. Cohen's *d*, Pearson's *r*), indicating how they were calculated

Our web collection on statistics for biologists contains articles on many of the points above.

Software and code

Policy information about availability of computer code

Data collection	The code used to analyze the single-cell and cfDNA genomics data uses standard third-party open-source libraries and packages in R and Python. The code used to identify CTCs in the HDSCA workflow uses custom code and is proprietary and licensed to Epic Sciences for commercial use.
Data analysis	Statistical two-sided analyses were performed using R (R-4.0.3., Boston, MA).

For manuscripts utilizing custom algorithms or software that are central to the research but not yet described in published literature, software must be made available to editors and reviewers. We strongly encourage code deposition in a community repository (e.g. GitHub). See the Nature Portfolio guidelines for submitting code & software for further information.

Data

Policy information about availability of data

All manuscripts must include a data availability statement. This statement should provide the following information, where applicable:

- Accession codes, unique identifiers, or web links for publicly available datasets
- A description of any restrictions on data availability
- For clinical datasets or third party data, please ensure that the statement adheres to our policy

All data discussed in this manuscript are either included in the main manuscript text or in the Supplementary Information Files. Some of the data can be accessed through our website http://pivot.usc.edu/. The sequencing data of the single cells and cfDNA is available through the BloodPAC Data Commons Accession ID "BPDC000119".

Field-specific reporting

Please select the one below that is the best fit for your research. If you are not sure, read the appropriate sections before making your selection.

☒ Life sciences ☐ Behavioural & social sciences ☐ Ecological, evolutionary & environmental sciences

For a reference copy of the document with all sections, see nature.com/documents/nr-reporting-summary-flat.pdf

Life sciences study design

All studies must disclose on these points even when the disclosure is negative.

Sample size	Five patients with metastatic BC from the MutHER or SUMMIT trials had PB samples collected for liquid biopsy analysis using the HDSCA workflow. Multiple draws were collected for each patient, allowing for a total of 24 PB samples to be evaluated.
Data exclusions	All samples were analyzed and data provided.
Replication	The single cell and cfDNA data here can not be reproduced as it is patient provided tissue.
Randomization	This is not relevant to our study. This was an observational study of liquid biopsy analytes detected in patients on trial.
Blinding	This is not relevant to our study. This was an observational study of liquid biopsy analytes detected in patients on trial.

Reporting for specific materials, systems and methods

We require information from authors about some types of materials, experimental systems and methods used in many studies. Here, indicate whether each material, system or method listed is relevant to your study. If you are not sure if a list item applies to your research, read the appropriate section before selecting a response.

Materials & experimental systems

n/a	Involved in the study	
☐	☒	Antibodies
☒	☐	Eukaryotic cell lines
☒	☐	Palaeontology and archaeology
☒	☐	Animals and other organisms
☐	☒	Human research participants
☐	☒	Clinical data
☒	☐	Dual use research of concern

Methods

n/a	Involved in the study	
☒	☐	ChIP-seq
☒	☐	Flow cytometry
☒	☐	MRI-based neuroimaging

Antibodies

Antibodies used	Slides were incubated with Alexa Fluor 647 conjugated anti-CD45 (1:125; clone: F10-89-4, MCA87A647, AbD Serotec, Raleigh NC, USA), anti-ER (1:250, clone SP1, MA5-14501clone SP1, 1:250, ThermoFisher, Waltham, MA, USA) followed by an Alexa Fluor 488 secondary (1:1000, A-11008, Invitrogen, Waltham, MA), and antibodies against cytokeratins (panCK, 1:100, clones: C-11, PCK-26, CY-90, KS-1A3, M20, A53-B/A2,C2562, Sigma, St. Louis MO USA; CK19, 1:100; , clone: RCK108, GA61561-2, Dako, Carpinteria, USA) followed by an Alexa Fluor 555 secondary (1:500, A21127, Invitrogen, Waltham, MA).
Validation	Overall performance, including sensitivity and specificity, in both healthy donors and cancer patients was described previously. References provided in text.

Human research participants

Policy information about studies involving human research participants

Population characteristics	We evaluated the clinical response and genomic profiles of five post-menopausal patients with metastatic ERBB2 mutant, non-amplified BC from the MutHER (NCT01670877) or SUMMIT (NCT01953926) trials being seen at the USC Norris Comprehensive Cancer Center. Patients on this study had an average of 5.4 lines of therapy before being placed on a combined treatment consisting of neratinib and fulvestrant. Eligibility required identification of somatic ERBB2 mutation with the absence of HER2 expression or ERBB2 amplification (0 or 1+ by immunohistochemistry or non-amplified by FISH). Patients received neratinib 240mg PO daily (28-day cycle) and 500mg fulvestrant (28-day cycle) with prophylactic loperamide to reduce the frequency of diarrhea.
Recruitment	During the treatment period, PB samples were collected at multiple timepoints as a liquid biopsy to molecularly characterize CTCs and cfDNA. Those patients with samples collected were included in this study.

Ethics oversight	Approved by the institutional review board of the University of Southern California's Keck School of Medicine (Los Angeles, California, United States).

Note that full information on the approval of the study protocol must also be provided in the manuscript.

Clinical data

Policy information about clinical studies

All manuscripts should comply with the ICMJE guidelines for publication of clinical research and a completed CONSORT checklist must be included with all submissions.

Clinical trial registration	Samples collected from patients on the MutHER (NCT01670877) or SUMMIT (NCT01953926) trials
Study protocol	Full study protocols are available via clinicaltrials.gov
Data collection	Data was collected during the treatment period and provided as de-identified information in a secure REDCap database.
Outcomes	This was an observational study without outcome measures pre-defined
